# A comprehensive analysis of the physiological and biochemical responses of raspberry cultivars to water stress

**DOI:** 10.1038/s41598-025-27434-7

**Published:** 2025-11-24

**Authors:** Duygu Ayvaz Sonmez, Azam Akbari, Betül Yeşil, Salih Kafkas, Bruno Mezzetti, Nesibe Ebru Kafkas

**Affiliations:** 1 Yaltır Agricultural Products Sarıhuğlar, Adana, Turkey; 2 Department of Horticulture, Faculty of Agriculture, University of Çukurova, 01330 Adana, Turkey; 3 Department of Agronomy and Plant Breeding Science, College of Aburaihan, University of Tehran, Tehran, Iran; 4 Department of Agricultural, Food and Environmental Sciences, Universita Politecnica delle, Ancona, Marche Italy

**Keywords:** Rubus idaeus, Water stress tolerance, Physiological traits, Biochemical markers, Biochemistry, Plant sciences

## Abstract

Drought tolerance mechanisms are crucial for global crop production under increasing water scarcity. It is important to understand these mechanisms in raspberry (*Rubus idaeus* L.) cultivars to support their water-limited stress tolerance. This study assessed the physiological, biochemical, and leaf morphological responses of two commercial cultivars, ‘Diamond Jubilee’ and ‘Jade’, across two seasons (2022 and 2024) under controlled irrigation: full irrigation (100%), moderate drought (50%), and PEG-induced osmotic stress in 2022 and two treatments (100% and PEG) in 2024. The responses were significantly influenced by both genotype and treatment. Under PEG stress conditions, ‘Jade’ maintained superior water status with RWC of 48.1% in 2022 and 66.7% in 2024 compared to ‘Diamond Jubilee’ (56.0% in 2022 and 32.4% in 2024), representing 37.4% reduction vs 63.8% reduction relative to their respective controls, indicating greater physiological stability. In contrast, ‘Diamond Jubilee’ showed stronger biochemical responses, with proline increasing from 0.037 to 0.114 μmol/g (1,171% increase) and peroxidase activity rising from 24.4 to 93.9 U/g/min (284.8% increase) in 2022, suggesting enhanced antioxidant defense through multiple enzymatic and non-enzymatic components. Both cultivars accumulated soluble sugars under drought stress, with glucose content increasing from 2.56 to 4.25% (66.0% increase) in 2022 and from 2.59 to 3.09% (19.5% increase) in 2024, indicating osmotic adjustment mechanisms. Total phenolic content increased from 432 to 620 mg GAE/100 g (43.6% increase) in 2024 under PEG treatment. Organic acid analysis in 2024 revealed cultivar-specific responses: citric acid increased from 4.41 to 7.10 mg/g DW (61.0% increase) in ‘Diamond Jubilee’ and from 2.83 to 3.77 mg/g DW (33.2% increase) in ‘Jade’, while ascorbic acid was completely depleted from 0.31 and 0.21 mg/g DW to 0.00 mg/g DW in both cultivars. Oxalic acid showed contrasting responses, increasing from 2.54 to 3.33 mg/g DW in ‘Diamond Jubilee’ but decreasing from 4.12 to 3.60 mg/g DW in ‘Jade’. Principal Component Analysis captured 77.1% of variance in 2022 and 90.2% in 2024, clearly separating cultivars and treatments. Based on superior water retention capacity and maintenance of photosynthetic efficiency across both years, ‘Jade’ demonstrated greater physiological resilience, while ‘Diamond Jubilee’ showed enhanced metabolic plasticity through active osmotic and biochemical stress responses. These findings highlight key traits—relative water content, proline accumulation, phenolic compounds, peroxidase activity, and cultivar-specific organic acid profiles—that can support drought-tolerant raspberry cultivar selection in breeding programs.

**Supplementary Information:**

The online version contains supplementary material available at 10.1038/s41598-025-27434-7.

## Introduction

Water scarcity is among the most pressing threats to global agricultural production, particularly under climate change and increasing food demands. By 2050, many historically productive agricultural regions are expected to face severe water deficits, potentially causing yield losses of 4–10%^1,2^. These projections stress the urgency of breeding and management interventions. To sustain production, developing drought-tolerant cultivars and adopting water-saving management practices are central goals in modern agriculture^3^. Raspberry (*Rubus idaeus* L.) is a high-value fruit crop valued for economic and nutritional attributes. Its fruits contain anthocyanins, ellagic acid, quercetin, and vitamin C—bioactive compounds with well-documented antioxidant, anti-inflammatory, and anticancer effects^4^. These properties have driven consumer demand and global cultivation. However, raspberries are highly sensitive to water deficits due to their shallow root system (25–30 cm) and high seasonal water requirements^5^. Even brief water shortages during critical phenological stages, such as fruit formation, can reduce yields within 7–10 days^6^. This highlights the need to understand mechanisms underpinning drought tolerance in this species.

Under water limitation, plants initiate a cascade of physiological and biochemical responses to maintain homeostasis. Stomatal closure is an early response, reducing transpiration and water loss but also limiting carbon assimilation^7^. In raspberry, drought decreases stomatal conductance, transpiration, and net photosynthesis, particularly when leaf water potential drops below critical thresholds^8,9^. The magnitude of these effects depends on the intensity, timing, and duration of stress, all influencing yield^10^. Physiological indicators of drought in raspberry include elevated leaf temperature from reduced transpirational cooling, decreased relative water content (RWC) indicating impaired water status, and reduced photosynthetic quantum yield reflecting damage or downregulation of photosystem II^11^. Moderate drought (40–60% field capacity) can lower maximum quantum efficiency (Fv/Fm) by 15–25%, reduce mesophyll conductance by 30–45%, and raise canopy temperature by 2–4 °C^12^. Such changes often coincide with morphological adaptations—smaller leaf area, higher leaf dry matter content, and thicker cuticles—that help reduce water loss while maintaining essential metabolic activity^13^. Biochemically, drought reprograms metabolic pathways, especially those involving carbohydrate metabolism and antioxidant defense. Osmoprotectants such as proline facilitate osmotic adjustment and protect cellular structures^14,15^. Soluble sugars—sucrose, glucose, and fructose—serve as osmolytes, energy sources, and signaling molecules regulating stress-responsive genes^16^. Drought can also shift organic acid profiles, such as citric and succinic acids, reflecting altered respiration and carbon allocation^17^.

Oxidative stress is a key consequence of drought, as reactive oxygen species (ROS) accumulate and damage lipids, proteins, and nucleic acids. Plants counter this with enzymatic and non-enzymatic antioxidant defenses. In raspberry, drought increases total phenolic content and the activity of enzymes such as peroxidase, catalase, and polyphenol oxidase^18,19^. Proteomic studies show greater abundance of antioxidant enzymes like superoxide dismutase, ascorbate peroxidase, and glutathione reductase under drought^20^, emphasizing their role in maintaining redox balance (Yalav et al., 2024).

Phytohormones, particularly abscisic acid (ABA), are central to drought signaling, regulating short-term physiological responses—such as stomatal closure, osmotic adjustment, and maintenance of leaf water status—and longer-term adaptive changes through stress-responsive gene expression^21,22^. In raspberry, ABA interacts with jasmonates, salicylic acid, and brassinosteroids in complex regulatory networks that coordinate drought adaptation^8^. However, cultivar differences in ABA sensitivity and hormone crosstalk remain poorly explored.

Polyethylene glycol (PEG) is often used experimentally to simulate drought by reducing osmotic potential without adding salinity stress. High-molecular-weight PEG (e.g., PEG 6000) cannot penetrate cell membranes, inducing water stress without direct toxicity^23^. Comparing PEG-induced stress with soil drying helps identify shared and distinct drought responses, revealing robust physiological and biochemical markers for breeding^24,25^. Despite raspberry’s importance and drought sensitivity, integrated studies assessing physiological and biochemical responses across cultivars are limited. Most focus on single traits, neglecting the interplay among water relations, photosynthesis, metabolic reprogramming, antioxidant defense, and hormonal signaling^26^. Moreover, differences in drought adaptation between floricanes and primocanes remain underexplored, though such insights could aid targeted breeding and management^27,28^. The two raspberry cultivars used in this study, ‘Diamond Jubilee’ and ‘Jade’, were selected for their commercial importance and desirable fruit attributes. ‘Diamond Jubilee’ exhibits high cane vigor, large and firm fruits, low acidity, and extended postharvest shelf life^29^, while ‘Jade’ is valued for its yield potential and fruit quality. To the best of our knowledge, no published studies have evaluated the drought stress responses of either cultivar, highlighting the novelty of this work. The objective of this study was to evaluate their physiological and biochemical responses to soil water deficit and PEG-induced osmotic stress across two growing seasons, integrating data on water relations, photosynthetic traits, primary metabolites, antioxidant components, and hormonal regulators to elucidate drought tolerance mechanisms and identify traits relevant for breeding and sustainable irrigation management.

## Materials and methods

### Experimental location

This study was conducted at the Yalex commercial greenhouse in Adana, Turkey (37.01° N, 35.23° E, Elevation: 41 m above sea level). The research took place in the YALTIR long greenhouse tunnel (YL-HT-GH), which is equipped with a partial environmental control system.

### Plant materials and growing conditions

Two commercial cultivars of red raspberry (*Rubus idaeus* L.), ‘Diamond Jubilee’ and ‘Jade’, were evaluated in this study. Uniform five-year-old raspberry plants were procured from a certified commercial nursery located in Adana, Türkiye, and transplanted into 5-L plastic pots. The growing substrate consisted of a mixture of peat moss, perlite, and vermiculite in a 2:1:1 ratio (v/v/v), with pH adjusted to 5.5–6.5, suitable for raspberry cultivation. Irrigation was applied regularly to maintain adequate moisture throughout the root zone. Plants were grown under greenhouse conditions. During the experimental periods, recorded greenhouse temperatures were 18–33 °C (mean 27.3 °C) in 2022 (October) and 29–38 °C (mean 34.0 °C) in 2024 (July ), reflecting seasonal differences. Relative humidity ranged from 60 to 70%. Lighting was primarily natural, supplemented with LED lamps to maintain a 16/8 h light/dark photoperiod. Before the application of drought treatments, all plants were acclimated for 4 weeks under well-watered conditions.

Plants were fertigated daily with a complete nutrient solution containing both macroelements (N, P, K) and microelements (Fe, Mn, Zn, Cu, B, Mo). The solution was maintained at a pH of 5.8–6.0 and an electrical conductivity (EC) of 1.3 dS/m.

### Experimental design and treatments

The study was conducted over two growing seasons (2022 and 2024) using a randomized complete block design (RCBD) with three replications per treatment. In 2022, three irrigation treatments were applied: full irrigation (100% field capacity), moderate drought (50% irrigation), and osmotic stress using 20% (w/v) Polyethylene Glycol 6000 (PEG). Based on the 2022 results—where the 50% irrigation treatment produced intermediate and less distinct physiological and biochemical responses—the 2024 experiment was simplified to include only the two most contrasting treatments: full irrigation and PEG-induced stress. No experimental data were collected in 2023, as the plant material was under propagation and preparation for the subsequent trial season.

Irrigation was managed to maintain consistent soil moisture targets verified by regular monitoring: 35–40% volumetric water content (field capacity) for 100% treatment, 18–23% for 50% treatment (2022 only), and -0.5 MPa osmotic stress for PEG treatment (20% w/v PEG 6000). In 2022, irrigation events during the establishment phase delivered approximately 240 ml (100% treatment) or 120 ml (50% treatment) per application. The 2024 experiment followed the same irrigation protocol with seasonal adjustments to maintain identical soil moisture targets.

Each experimental unit included one single plant per plot, giving a total of 18 plants in 2022 (2 cultivars × 3 treatments × 3 replications) and 12 in 2024 (2 cultivars × 2 treatments × 3 replications). Although four cultivars were initially planted for broader evaluation, only these two were subjected to detailed physiological and biochemical analyses.

### Physiological parameters

#### Leaf temperature

Leaf temperature measurements were conducted using a Fluke 62 Max handheld infrared thermometer. This device allows for non-contact measurement of leaf surface temperature by detecting emitted thermal radiation. The measurements were conducted during the morning hours, typically between 08:00 and 10:00, to avoid the midday heat and reduce variations due to direct sunlight exposure. The infrared thermometer was positioned approximately 0.5 m above the plant canopy and aimed directly at the leaves to obtain accurate readings. Each leaf was measured three times to ensure consistency, and the average temperature was recorded. This method is particularly useful for assessing the physiological responses of blueberry plants under various environmental conditions, including drought stress (González-Villagra et al., 2024; Barai et al., 2025).

#### Leaf relative water content (RWC)

The relative water content (RWC) was determined according to Turner (1981) with modifications by Wilkinson et al. (2001). The leaves were freshly detached from the plant (approx. 1 g) for weighing to record fresh weight (FW), and then submerged in a 25 ml beaker of water, in the dark for one night. The following day leaves were weighed again for turgid weight (TW) before they were dried in an oven at 80°C for 24 h to determine dry weight (DW). Relative Water Content was calculated using the following formula: RWC (%) = [(FW–DW) / (TW–DW)] × 100.

#### Chlorophyll content

The non-destructive estimation of leaf chlorophyll content utilized a chlorophyll meter (SPAD-502 Plus, Konica Minolta, Japan). Five measurements were made per leaf (to prevent measuring over major veins) on five leaves per plant and averaged. SPAD values were converted into mmol m-2 of chlorophyll content using the following calibration equation, developed by Markwell et al. (1995), which converts SPAD values to chlorophyll content: Chlorophyll content = 10^(SPAD × 0.0265 + 0.9).

#### Photosynthetic quantum yield (ΦPSII)

Chlorophyll fluorescence was measured using a portable fluorometer (MINI-PAM-II, Walz, Germany) following Baker . Measurements were performed on five fully expanded leaves per plant (three plants per treatment) from the middle section of the shoots, between 08:00 and 10:00 h to avoid potential effects of midday photoinhibition. Leaf temperature during measurements ranged from 20 to 23 °C in 2022 and from 28–32 °C in 2024. The effective quantum yield of PSII (ΦPSII) was calculated as (Fm′ – Fs)/Fm′, where Fs is steady-state fluorescence and Fm′ is maximum fluorescence under actinic light. Lower ΦPSII values were interpreted as an indication of greater stress impact on photosynthetic efficiency (Maxwell & Johnson, 2000).

#### Gas exchange measurement

Gas exchange measurements were performed only in the 2022 experiment. Measurements were made at harvest time with a portable infrared gas analyzer (IRGA) (Li-6400; LI-COR, Inc., Lincoln, NE, USA), as reported by Reyes-Díaz et al. (2011). The following parameters were recorded: CO2 assimilation (Pn), stomatal conductance (gs), transpiration (E), and intercellular CO2 concentration (Ci). The CO2 concentration standard was 400 μmol mol^−1^, the flow rate was 300 mL min^−1^, and 60% relative humidity in the leaf chamber, and the temperature was controlled at 20 ± 2 °C. The measurements were conducted on attached fully expanded leaves from 08:00 to 10:00 h in natural light. Five measurements were conducted on each of the plants.

### Biochemical parameters

#### Preparation of the extracts

For biochemical assays involving stable metabolites, fully expanded leaves were oven-dried at 60 °C until constant weight and ground into a fine powder. Unless otherwise specified in the individual assay protocols, 0.5 g of powdered tissue was extracted with 2.5 mL of 80% (v/v) methanol in 15 mL centrifuge tubes. The mixture was vortexed thoroughly and centrifuged at 4000 g for 10 min at 4 °C, and the resulting supernatant was collected and used for subsequent biochemical analyses, including total phenolic content, soluble sugars, and organic acids. Fresh leaf tissue was used separately for enzyme activity assays (PPO, POD) and ABA determination to preserve protein integrity and hormone stability. All analyses were performed with three biological replicates.

#### Sugars determination

Sucrose, glucose, fructose, and total sugar content were analyzed by an HPLC (Shimadzu LC 20A VP, Kyoto, Japan) with a refractive index detector. A reverse-phase Ultrasphere Coregel-87 C column, 300 mm × 7.8 mm, 5 µm at 70 °C, ultrapure water as the mobile phase at 0.6 mL/min under isocratic conditions was employed to separate them. A sample analysis of 20 µL was injected. The concentrations of sugars were measured with the aid of standard calibration and expressed as a percentage of dry weight (DW).

#### Determination of total phenolics

The total phenolic content of leaf extracts was measured using the Folin–Ciocalteu (FC) method (Singleton et al., 1999). Briefly, 0.1 mL of extract was mixed with 0.1 mL of FC reagent and 0.9 mL of water. After 5 min, 1 mL of 7% Na₂CO₃ and 0.4 mL of water were added. The mixture was incubated for 30 min, and absorbance was recorded at 765 nm. A blank solution (water + reagents) was used for calibration. Results were expressed as mg gallic acid equivalents (GAE) per 100 g of leaf dry weight, based on a standard curve. Analyses were performed in triplicate.

#### Organic acid determination

Individual organic acid determination was performed only in the 2024 experiment. Leaf tissue samples were used for organic acid analysis. Quantification of L-ascorbic acid, citric acid, and succinic acid was carried out on an HPLC (Shimadzu LC-20AD, Kyoto, Japan) with a diode array detector (Shimadzu SPD-20A VP) and an 87 H analytical column (5 µm, 300 mm × 7.8 mm). Chromatographic separation was conducted at 40 °C using 0.05 mM sulfuric acid as the mobile phase under isocratic conditions. The system used a 20 µL injection volume, 210 nm detection wavelength, and a flow rate of 0.8 mL/min. Organic acids were identified by retention time and spectral similarity to standards and quantified using standard calibration curves. Results were expressed as a percentage of dry weight (% DW).

#### Stress-related compounds

##### Proline estimation

To conduct the proline assay according to Bates et al. (1973), 0.2 g of leaf tissue was homogeneously mixed in a volume of 3 ml of 3% (w/v) sulfosalicylic acid. We softly rotated the tube to break up and homogenize the leaf tissue before centrifugation for 15 min at 18,000 × g. Two ml of the supernatant was transferred to a new tube after centrifugation, then 2 ml of glacial acetic acid and 2 ml of the ninhydrin reagent were added to this new tube. The mixtures were placed in a 100 °C water bath for 1 h, and then cooled down in an ice bath for 5 min. Subsequently, 4 ml (volume) of toluene was added to stop the reaction, and the absorbance was recorded at 520 nm of the toluene phase using a spectrophotometer.

##### Abscisic acid (ABA) quantification

ABA extraction was performed according to the procedure of Huang et al. (2014) with some modifications. Fresh leaf tissues (0.5 g) were extracted with 5 mL of 80% methanol including 1% acetic acid and 10 ng internal standard (^2^H_6_-ABA). Following centrifugation (10,000 g, 15 min, 4°C), the supernatant was collected, dried completely, and re-dissolved in 1 mL of 1% acetic acid. The extract was then purified through a C18 solid-phase extraction cartridge. ABA was measured by HPLC (Shimadzu LC-20AD system, diode array detector (Shimadzu SPD-20A VP), and 87 H analytical column (5 µm, 300 mm × 7.8 mm i.d., Transgenomic)). Results were presented as ng/g fresh weight.

#### Enzyme activity (PPO and POD)

##### Polyphenol oxidase (PPO) activity

PPO activity was measured following the Aquino-Bolaños and Mercado-Silva (2004) procedure. 1 g of leaf tissue was ground to a fine powder in 10 mL 0.1 M phosphate buffer pH 6.5 containing 1% polyvinylpyrrolidone and 0.1 M EDTA and centrifuged at 15,000 g for 20 min at 4°C. The supernatant was employed as the enzyme extract. PPO activity was determined by mixing 0.1 mL extract with 2.9 mL of 0.1 M catechol solution in phosphate buffer (pH 6.5) and reading at 420 nm for 3 min. Values were expressed as change in absorbance per minute per gram fresh weight.

##### Peroxidase (POD) activity

POD activity was determined according to Chance and Maehly (1955). Reaction mixture comprised 0.1 mL enzyme extract, 2.8 mL 0.1 M pH 6.5 phosphate buffer, and 0.1 mL 1% H2O2, and reaction was initiated by the addition of 0.1 mL 4% guaiacol. Absorbance increase at 470 nm was recorded for 3 min, and activity was expressed as increase in absorbance per minute per gram fresh weight.

#### Leaf morphological parameters

Leaf samples were collected from fully expanded, non-senescent leaves (mid-mature stage), i.e., leaves that were neither newly emerged nor aged. To ensure consistency, leaves with similar morphological characteristics were selected from each plant. Leaf area was then determined by scanning the fully expanded leaves using a flatbed scanner (Epson Perfection V800, Epson America Inc.) and analyzing the images using Digimizer image analysis software (MedCalc Software Ltd, Ostend, Belgium). Five leaves were measured per plant, and the area was expressed as cm^2^ per leaf.

Leaf length were measured on five leaves per plant using a digital caliper (Mitutoyo 500-196-30, Mitutoyo Corporation, Kawasaki, Japan) with an accuracy of 0.01 mm. Both leaf area and leaf length were assessed only in the 2022 experiment.

For Leaf Dry Matter Content (LDMC) measurements, leaves were collected separately from each plant. Fresh leaves were weighed immediately after collection to determine fresh weight (FW). The samples were then dried in an oven at 70 °C for 72 h, or until a constant weight was achieved, and reweighed to determine dry weight (DW). Leaf dry matter content (LDMC) was calculated as (DW / FW) × 100%.

#### Statistical analysis

Statistical analyses were performed using SAS software (v9.4, SAS Institute Inc., USA). Due to differences in irrigation treatments across years, separate two-way ANOVAs were conducted for 2022 and 2024, with cultivar, treatment, and their interaction as fixed effects. A combined three-way ANOVA was also performed to assess overall effects of year, cultivar, and treatment. Tukey’s HSD test (*p* < 0.05) was used for mean comparisons. Assumptions of normality and homogeneity of variances were tested and met.

Principal component analysis (PCA) and hierarchical clustering with heatmap visualization were conducted in R (v4.4.3) to explore multivariate patterns among cultivars.

## Results and discussion

### Physiological responses

#### Leaf temperature response

**Fig. 1:**
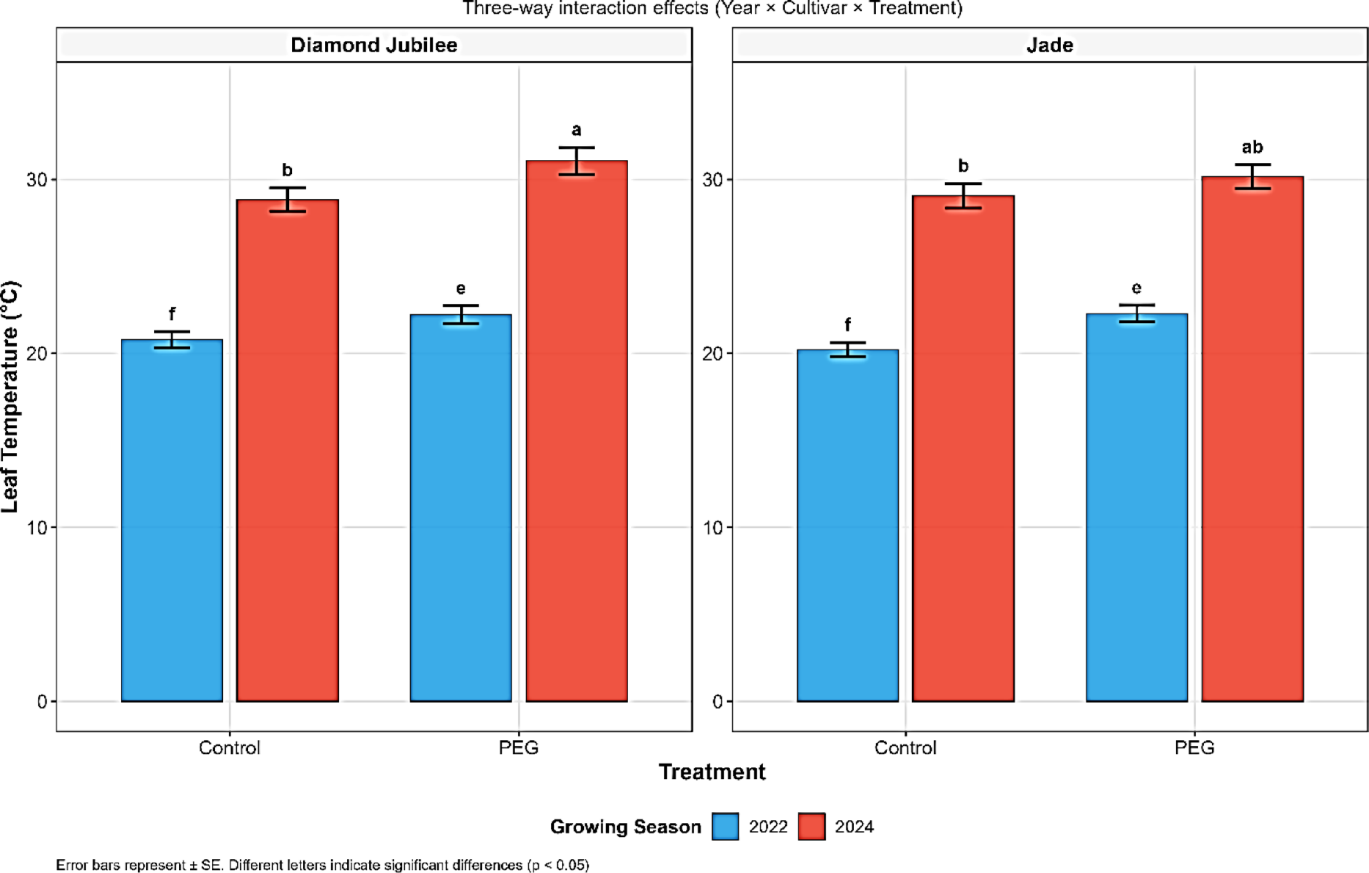
Three-way interaction effects (Year × Cultivar × Treatment) on leaf temperature (°C) of raspberry cultivars ‘Diamond Jubilee’ and ‘Jade’ under control and PEG-induced drought stress conditions across two growing seasons (2022 and 2024).

The combined ANOVA showed significant effects of treatment (F = 17.51, *P* < 0.001) and year × treatment interaction (*P* < 0.05) on leaf temperature (Table 1). PEG stress increased leaf temperature by 6.9% compared with controls (26.45 ± 0.67 °C vs. 24.74 ± 0.45 °C; Table 2, Fig. 1), with Diamond Jubilee showing greater temperature sensitivity (28.85–31.08 °C) than Jade (29.07–30.17 °C) in 2024 (Table S3).

**Table 1:** Analysis of variance for physiological, biochemical, and stress-related traits in two raspberry cultivars (Diamond Jubilee and Jade) subjected to water deficit treatment (100% and PEG) treatments across two growing seasons (2022–2024).

Source	Year	Cultivar	Year × Cultivar	Treatment	Year × Treatment	Cultivar × Treatment	Year × Cultivar × Treatment
DF	1	1	1	1	1	1	1
Leaf Temp (°C)	423.19***	0.53ns	17.51***	0.01ns	0.01ns	0.09ns	1.17*
RWC (%)	13.07ns	508.52***	8775.49***	328.46**	7.06ns	95.99ns	1124.81***
Chlorophyll (µmol/m^2^)	302.46***	28.02***	76.09***	0.02ns	4.98ns	28.60***	4.86ns
Photosynthetic quantum yield of leaf	0.04***	0.02***	0.01***	0.01***	0ns	0ns	0ns
Dried leaf sucrose content (%)	9.99***	0.14ns	1.58***	3.76***	0.45*	0.12ns	0.02ns
Dried leaf glucose content (%)	7.70***	0.06ns	17.26***	1.90**	8.47***	0.14ns	3.34**
Dried leaf fructose content (%)	0.19ns	0.26ns	12.77***	0.87*	0.61ns	0ns	0.23ns
Dried leaf total sugar (%)	0ns	1.71ns	70.66***	12.26**	14.25**	0.01ns	3.24ns
Total phenol (mg GAE/100g)	126,466.69***	3417.45ns	72,299.19***	76.67ns	36,769.44***	7238.58*	15,772.86**
Proline (μmol/g)	0.72***	0.98***	3.30***	2.01***	0.67***	1.02***	2.06***
ABA (ng/g)	18.32***	5.77*	302.53***	0.02ns	8.97**	68.85***	3.98ns
POD (U/g/min)	28.38ns	18.90ns	11,655.63***	358.05**	1292.13***	151.50*	1493.10***
PPO (U/g/min)	45,937.5***	1768.17***	31,392.67***	16,642.67***	16,120.17***	4428.17***	7210.67***
Leaf dry matter content (%)	174.18*	15.45ns	1562.74***	44.35ns	8.34ns	12.65ns	22.46ns

Significance levels: ***P < 0.001, **P < 0.01, *P* < *0.05, ns* = *not significant.*

**Table 2:** Main effects of year (2022–2024), cultivar (Diamond Jubilee and Jade), and water deficit treatment (100% and PEG) on physiological and biochemical responses in raspberry plants based on combined analysis.

Factor	Level	Water relations		Photosynthetic performance		Osmoregulators		Antioxidant defense	
		Leaf temperature (°C)	RWC (%)	Chlorophyll (µmol/m^2^)	Quantum yield	Proline (μmol/g)	ABA (ng/g)	POD (U/g/min)	PPO (U/g/min)
Year	2022	21.39 ± 0.32 b	70.67 ± 3.45 a	33.93 ± 1.12 b	0.77 ± 0.01 a	0.10 ± 0.08 b	92.79 ± 1.23 b	57.31 ± 4.21 a	120.25 ± 8.45 b
	2024	29.79 ± 0.41 a	69.93 ± 4.12 a	41.03 ± 1.08 a	0.69 ± 0.02 b	0.22 ± 0.03 a	94.53 ± 1.45 a	58.35 ± 3.89 a	207.75 ± 12.32 a
Cultivar	Diamond Jubilee	25.71 ± 0.89 a	65.33 ± 3.12 b	36.39 ± 1.01 b	0.75 ± 0.01 a	0.19 ± 0.04 a	93.17 ± 1.12 b	56.23 ± 3.45 a	172.58 ± 9.87 a
	Jade	25.47 ± 0.76 a	74.54 ± 3.89 a	38.56 ± 1.23 a	0.70 ± 0.02 b	0.12 ± 0.09 b	94.15 ± 1.34 a	59.43 ± 4.12 a	155.42 ± 8.23 b
Treatment	100%	24.74 ± 0.45 b	89.06 ± 2.34 a	39.26 ± 1.12 a	0.75 ± 0.01 a	0.02 ± 0.01 b	90.11 ± 1.01 b	35.23 ± 2.12 b	200.17 ± 11.23 a
	PEG	26.45 ± 0.67 a	50.81 ± 2.89 b	35.69 ± 1.01 b	0.71 ± 0.02 b	0.29 ± 0.08 a	97.21 ± 1.45 a	79.30 ± 4.56 a	127.83 ± 7.89 b

Values represent means ± SE. Different letters within each factor indicate significant differences (*P* ≤ 0.05, Tukey’s HSD test).

Leaf temperature increases under drought directly reflect stomatal closure, which limits transpiration and reduces evaporative cooling^30^. When stomata are open, transpiration cools the leaves, but as drought stress progresses and stomata close, leaf temperature increases due to reduced latent heat exchange, making leaf temperature a reliable proxy for transpiration and water status^31^. The rate of transpiration is inversely proportional to leaf temperature, allowing early detection of drought stress before visual symptoms appear (Münchinger et al., 2024). Cultivar differences suggest that Diamond Jubilee adopts a more conservative stomatal strategy compared to Jade‘s better maintenance of cooling capacity^32^, highlighting thermal regulation as an integrated component of gas exchange responses in raspberry drought tolerance^33^.

#### Relative water content

**Fig. 2:**
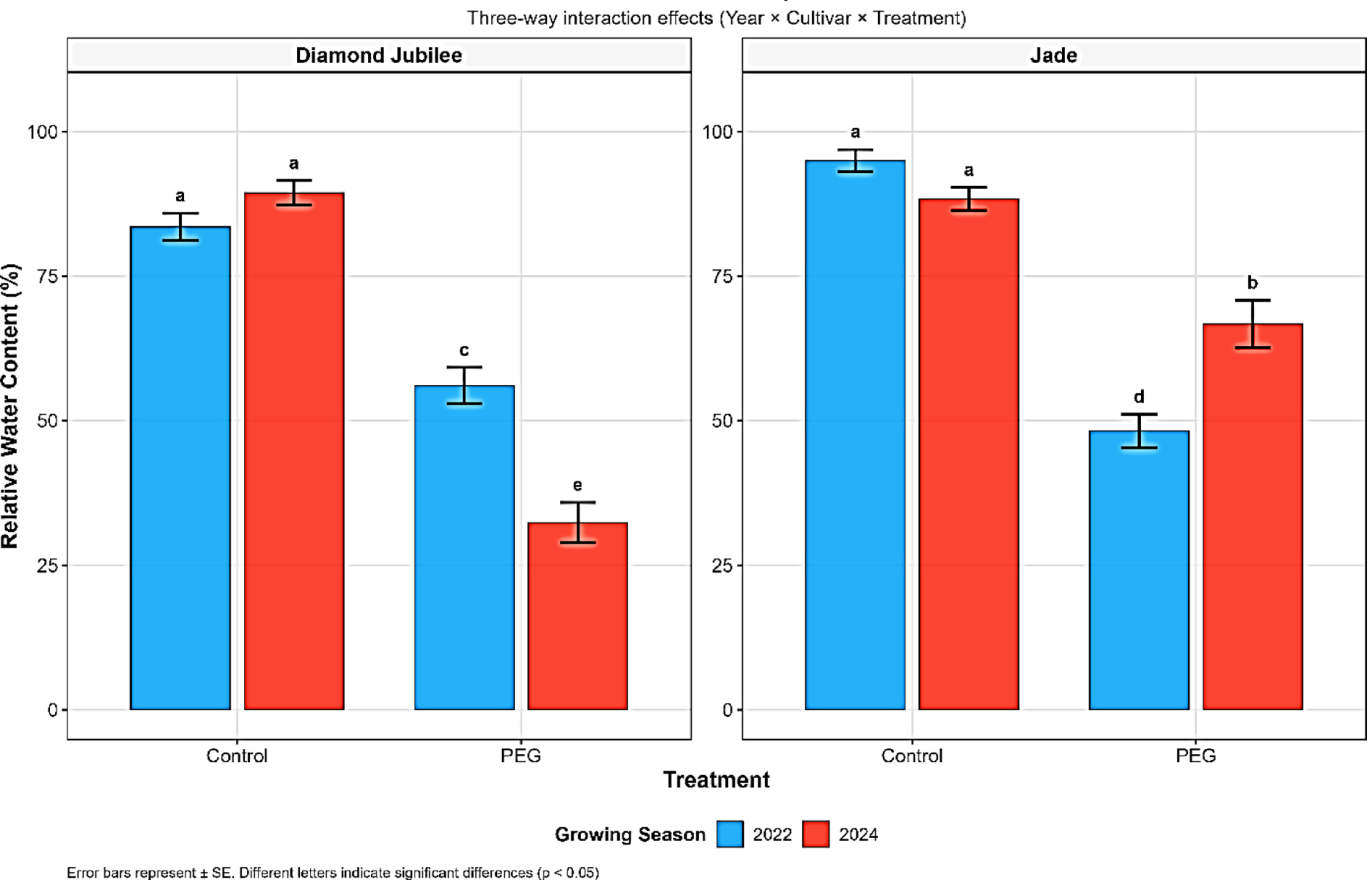
Effects of Year × Cultivar × Treatment interaction on relative water content (RWC, %) of raspberry cultivars ‘Diamond Jubilee’ and ‘Jade’ under control and PEG-induced water stress conditions during the 2022 and 2024 growing seasons.

Analysis of variance showed highly significant effects of cultivar and treatment on RWC, with a strong year × cultivar × treatment interaction (*P* < 0.001, Table 1, Fig. 2), confirming RWC as the most drought-sensitive parameter. Across treatments, ‘Jade’ consistently maintained superior water status (74.5%) compared with ‘Diamond Jubilee’ (65.3%), while PEG stress caused the most severe reduction (from 89.1 to 50.8%). Genotypic differences were evident, as ‘Diamond Jubilee’ lost nearly half of its RWC, whereas ‘Jade’ retained more water with only a 37.4% reduction. These findings support earlier reports identifying water status as a reliable indicator of drought tolerance^34,35^.

The enhanced performance of ‘Jade’ can be attributed to stronger osmotic adjustment and aquaporin regulation. Aquaporins are crucial for water transport but are often downregulated under drought to limit water loss^36,37^, while solute accumulation helps sustain turgor and photosynthesis^38^. Temporal responses revealed cultivar-specific adaptation: from 2022 to 2024, ‘Jade’ improved its acclimation capacity (49.3–24.5% reduction), whereas ‘Diamond Jubilee’ became progressively more vulnerable (32.9–63.8%). Such contrasting responses may reflect stress memory and post-translational regulation of aquaporins^39^.

Moderate stress (50% irrigation) led to only limited RWC reductions (2.7% in ‘Diamond Jubilee’ and 18.1% in ‘Jade’), suggesting threshold-dependent effects (Table S6). These patterns are consistent with findings on genotype-dependent drought physiology in raspberry^40,41^ and with recent grapevine studies that identified RWC as a sensitive drought indicator^42^. Overall, the results highlight superior drought acclimation in ‘Jade’, emphasizing its potential value for breeding and management under water-limited conditions.

#### Chlorophyll content

Analysis of variance revealed significant effects of year (*P* < 0.001), cultivar (*P* < 0.001), and treatment (*P* < 0.001) on chlorophyll content, with notable cultivar × treatment interactions (*P* < 0.001, Table 1). Chlorophyll content was consistently higher in 2024 (41.03 ± 1.08 µmol/m^2^) compared to 2022 (33.93 ± 1.12 µmol/m^2^), while ‘Jade’ maintained superior chlorophyll levels (38.56 ± 1.23 µmol/m^2^) over ‘Diamond Jubilee’ (36.39 ± 1.01 µmol/m^2^) across treatments (Table 2).

Water deficit significantly reduced chlorophyll content from 39.26 ± 1.12 µmol/m^2^ under optimal irrigation to 35.69 ± 1.01 µmol/m^2^ under PEG treatment (Table 2). The observed reduction in chlorophyll content under water stress is primarily attributed to chlorophyll degradation directly caused by drought, since water deficit both suppresses biosynthesis and accelerates degradation processes^43,44^. Cultivar-specific responses further highlighted this trend: ‘Diamond Jubilee’ experienced only a minimal reduction (3.7%) from 37.09 ± 1.23 to 35.71 ± 1.01 µmol/m^2^, while ‘Jade’ showed greater sensitivity with a 13.9% decline from 41.43 ± 1.45 to 35.68 ± 1.12 µmol/m^2^ under water stress (Table 3). These findings are in line with reports that different plant species and cultivars exhibit varying abilities to maintain chlorophyll stability under drought conditions^45^. The annual analysis (Table S2) confirmed these cultivar-dependent differences, with significant cultivar × treatment interactions in 2024 (*P* < 0.01) but not in 2022, reflecting temporal variation in stress responses. Such variation is consistent with the findings of Lepaja et al. (2020) on raspberry drought sensitivity. The supplementary three-level drought gradient (Table S4) further demonstrated that moderate stress (50% irrigation) did not significantly impact chlorophyll biosynthesis, corroborating Zahidi et al.^46^ who observed a similar trend in strawberry. This resilience under moderate stress suggests activation of adaptive mechanisms, such as antioxidant defense and photoprotective responses, whereas severe stress overwhelms these systems and leads to accelerated pigment degradation^47^.

**Table 3:** Cultivar-specific physiological and biochemical responses to water deficit treatment: Genotype × treatment interactions in raspberry plants based on combined analysis over two years (2022–2024).

Cultivar	Treatment	Photosynthetic System		Water status		Stress signaling		Enzymatic defense	
		chlorophyll (µmol/m^2^)	quantum yield	RWC (%)	Dry matter (%)	Proline (μmol/g)	ABA (ng/g)	POD (U/g/min)	PPO (U/g/min)
Diamond Jubilee	100%	37.09 ± 1.23 b	0.77 ± 0.01 a	86.45 ± 2.34 a	27.19 ± 2.01 c	0.028 ± 0.003 b	87.93 ± 1.12 c	36.85 ± 2.45 c	222.34 ± 15.23 a
	PEG	35.71 ± 1.01 b	0.74 ± 0.02 b	44.21 ± 3.12 c	44.39 ± 3.12 a	0.356 ± 0.045 a	98.41 ± 1.67 a	75.90 ± 4.23 b	122.82 ± 9.87 c
Jade	100%	41.43 ± 1.45 a	0.73 ± 0.02 b	91.66 ± 1.89 a	26.72 ± 1.89 c	0.018 ± 0.002 d	92.30 ± 1.34 b	33.60 ± 2.87 c	178.00 ± 12.45 b
	PEG	35.68 ± 1.12 b	0.68 ± 0.03 c	57.42 ± 2.89 b	40.51 ± 2.89 b	0.230 ± 0.089 ab	96.01 ± 1.89 a	82.70 ± 4.56 a	132.84 ± 8.67 c

Values represent means ± SE. Different letters indicate significant differences across all treatment combinations (P ≤ 0.05, Tukey’s HSD test). Only parameters showing significant cultivar × treatment interactions are presented.

Finally, the increased chlorophyll content in the second year likely reflects enhanced photosynthetic capacity associated with plant maturity, which aligns with the observations of Williams et al.^48^ on raspberry physiological development under stress conditions.

#### Photosynthetic quantum yield

Analysis of variance revealed significant effects of year (*P* < 0.001), cultivar (*P* < 0.001), and treatment (*P* < 0.001) on photosynthetic quantum yield, along with significant year × treatment interactions (*P* < 0.001; Table 1). Overall, values were higher in 2022 (0.77 ± 0.01) compared to 2024 (0.69 ± 0.02), and ‘Diamond Jubilee’ maintained superior performance (0.75 ± 0.01) relative to ‘Jade’ (0.70 ± 0.02) across treatments (Table 2). Water deficit reduced photosynthetic quantum yield from 0.75 ± 0.01 under full irrigation to 0.71 ± 0.02 under PEG treatment (Table 2). This decline reflects the high susceptibility of PSII to drought stress, where water deficit compromises PSII stability and efficiency in light energy conversion^49^.

Cultivar-specific responses further highlighted these differences: ‘Diamond Jubilee’ exhibited only a 3.9% reduction (0.77 ± 0.01 to 0.74 ± 0.02), while ‘Jade’ showed greater sensitivity with a 6.8% decline (0.73 ± 0.02 to 0.68 ± 0.03) under PEG stress (Table 3). These genotypic differences suggest a reconfiguration of the photosynthetic machinery under drought, involving modification of enzyme stoichiometry and phosphorylation of PSII and LHCII, which may vary among cultivars^50^.

Year-specific analyses also revealed temporal variation in stress responses. In 2022, reductions were modest (‘Diamond Jubilee’: 3.8%; ‘Jade’: 6.3%), whereas in 2024, the responses diverged: ‘Diamond Jubilee’ maintained stability with only a 1.4% reduction, while ‘Jade’ declined sharply by 8.9% under PEG stress (Table S6). These year × treatment interactions (Tables 4, S3) emphasize that photosynthetic responses are influenced by environmental variation between growing seasons, reflecting dynamic acclimation strategies. Such seasonal differences align with previous findings that drought adaptation integrates stomatal regulation and PSII non-photochemical quenching mechanisms to balance water conservation and excess energy dissipation, depending on environmental context^51^.

**Table 4:** Year-dependent metabolic responses to water deficit treatment in raspberry plants: Temporal dynamics of stress adaptation showing significant year × treatment interactions.

Year	Treatment	Soluble carbohydrates		Secondary metabolites	Osmotic adjustment	Hormonal regulation
		Glucose (%)	Total sugar (%)	Total phenolics (mg GAE/100 g)	Proline (μmol/g)	ABA (ng/g)
2022	100%	2.56 ± 0.12 c	9.61 ± 0.45 c	398.49 ± 12.34 b	0.029 ± 0.003 b	89.85 ± 1.23 b
	PEG	4.25 ± 0.23 a	13.04 ± 0.67 a	363.09 ± 15.67 b	0.164 ± 0.089 b	95.73 ± 1.67 a
2024	100%	2.59 ± 0.15 c	10.12 ± 0.34 c	431.95 ± 18.23 b	0.017 ± 0.002 b	90.37 ± 1.45 b
	PEG	3.09 ± 0.18 b	11.95 ± 0.56 b	619.99 ± 24.56 a	0.424 ± 0.045 a	98.69 ± 1.89 a

Values represent means ± SE. Different letters indicate significant differences across all treatment combinations within and between years (P ≤ 0.05, Tukey’s HSD test). Only parameters with significant year × treatment interactions are shown.

The sustained light-harvesting ability of ‘Diamond Jubilee’ under stress is consistent with the compensatory hydraulic and biochemical mechanisms reported in red raspberries^52^. In contrast, the higher sensitivity of ‘Jade’ resembles patterns observed in other berries, where water stress decreases PSII quantum yield but simultaneously induces protective defense mechanisms^53^. Together, these results highlight cultivar-dependent photosynthetic resilience and confirm the importance of genotype-specific adaptation strategies for maintaining productivity under drought^5^.

### Biochemical responses

#### Sugar content and composition

ANOVA results demonstrated significant treatment effects (*P* < 0.001) for total sugar content and individual sugar components, with significant year × treatment interactions indicating temporal variations in carbohydrate metabolism under water deficit stress (Table 1). Individual year analyses (Tables S1, S2) confirmed consistent treatment responses across both growing seasons, though with varying effect magnitudes.

Water deficit treatment (PEG) significantly increased total sugar content compared to control conditions (100% irrigation) across both years and cultivars (Table 4). The magnitude of this response varied between years, with PEG treatment increasing total sugar content by 35.7% in 2022 (from 9.61 to 13.04%) and by 18.1% in 2024 (from 10.12 to 11.95%). Similarly, sucrose content showed consistent increases under drought stress across both years (Table S5).

Individual sugar components showed differential responses to water deficit stress. Glucose content increased significantly under PEG treatment in both years, with greater accumulation observed in 2022 (66.0% increase) compared to 2024 (19.5% increase) (Table 4). Sucrose content exhibited significant year and treatment effects, while fructose content responded primarily to treatment effects with significant increases under water deficit conditions. Main effects analysis (Table 2) revealed no significant differences between cultivars for most carbohydrate parameters due to non-significant cultivar × treatment interactions.

The enhanced accumulation of soluble sugars under water deficit conditions serves multiple physiological functions in drought stress adaptation. These compatible solutes facilitate osmotic adjustment, enabling plants to maintain turgor pressure and continue essential metabolic processes during water stress^54^, with specific metabolic responses including glucose and fructose accumulation under PEG-induced stress^55^. The preferential accumulation of glucose and fructose during drought stress may be derived from starch degradation, and the increment in hexoses concentration provides osmotic protection^56^.

The differential sugar accumulation patterns between years provide evidence for stress memory mechanisms in raspberry plants. Plants hold on to past events in a way that adjusts their response to new challenges without altering their genetic constitution, enabling training for future stress events^57^. Stress priming through exposure to primary stress prepares plants to be more responsive to reoccurring stress through coordinated changes at organismal, cellular, and various omics levels^58^. The reduced carbohydrate responsiveness observed in 2024 compared to 2022 reflects adaptive metabolic efficiency following stress memory formation. This phenomenon involves epigenetic modifications that alter gene expression in carbohydrate metabolism pathways, allowing plants to optimize resource allocation during repeated stress events^59^. Epigenetic and chromatin-based mechanisms provide the molecular basis for environmental stress adaptation and stress memory in plants^60^. This adaptive plasticity allows plants to achieve similar drought protection with optimized resource allocation, demonstrating the practical importance of stress memory in perennial crops where repeated stress exposure is common.

#### Phenolic content

Analysis of variance revealed highly significant year × treatment interactions (*P* < 0.01) for total phenolic content (Table 1). Individual year analysis confirmed significant treatment effects with enhanced responses in the second year (Tables S1, S2).

Temporal analysis showed contrasting patterns between years (Table 4). In 2022, PEG treatment (363.09 ± 15.67 mg GAE/100 g) did not differ significantly from controls (398.49 ± 12.34 mg GAE/100 g). However, in 2024, a marked stress response was observed, with PEG-treated plants accumulating 619.99 ± 24.56 mg GAE/100 g compared to 431.95 ± 18.23 mg GAE/100 g in controls, representing a 43.6% increase.

The stronger response in the second year suggests stress memory mechanisms**,** where previous drought exposure primed phenolic biosynthesis pathways. Drought-induced phenolic production is primarily regulated via the phenylpropanoid biosynthetic pathway, in which numerous genes are modulated under water deficit^61^. This response often occurs in coordination with other adaptive mechanisms, including proline accumulation, ABA elevation, and enhanced antioxidant enzyme activities (Table S3). Phenolic compounds are well recognized for their role in stress adaptation, increasing under drought and contributing to plant tolerance^62^. Similar cultivar-dependent responses to water stress in raspberry were reported by Morales et al.^35^, while Efrose et al.^63^ demonstrated drought regulation of phenolic biosynthesis transcripts. The year-to-year and cultivar-dependent variation observed here supports the view that secondary metabolite biosynthesis is strictly controlled and highly responsive to environmental stress^64^. Moreover, controlled drought imposition has been successfully used to enhance bioactive compound content, particularly flavonoids, which play central roles in neutralizing ROS and protecting plants from oxidative damage^65,66^. This temporal enhancement thus reflects complex phenolic–adaptation interactions and highlights the potential of stress management as a tool to improve fruit quality.

#### Stress-related compounds

##### Proline content

Analysis of variance demonstrated highly significant effects of all main factors and their interactions on proline accumulation (*P* < 0.001 for all sources, Table 1). Proline content was markedly higher in 2022 (0.57 ± 0.08 μmol/g) compared to 2024 (0.22 ± 0.03 μmol/g), while ‘Jade’ accumulated significantly more proline (0.60 ± 0.09 μmol/g) than ‘Diamond Jubilee’ (0.19 ± 0.04 μmol/g) across treatments (Table 2). Water deficit stress induced dramatic proline elevation, rising from 0.02 ± 0.01 μmol/g under full irrigation (control) to 0.76 ± 0.08 μmol/g under severe drought stress imposed by PEG treatment, representing a 3,700% increase (Table 2).

Cultivar-specific responses revealed distinct osmotic adjustment strategies. ‘Diamond Jubilee’ showed moderate proline accumulation from 0.028 ± 0.003 to 0.356 ± 0.045 μmol/g (1,171% increase), while ‘Jade’ exhibited exceptional accumulation from 0.018 ± 0.002 to 1.173 ± 0.089 μmol/g (6,417% increase) under drought stress (Table 3). The three-way interaction analysis (Table S3) revealed temporal variation in stress responses, with 2022 showing more pronounced proline accumulation in ‘Jade’ (2.095 ± 0.089 μmol/g under severe PEG-induced stress) compared to 2024 (0.250 ± 0.023 μmol/g). Conversely, ‘Diamond Jubilee’ demonstrated increased proline responsiveness in 2024 (0.598 ± 0.045 μmol/g) compared to 2022 (0.114 ± 0.012 μmol/g under PEG treatment).

The supplementary gradient analysis (Table S4) confirmed dose-dependent proline responses in 2022, with moderate drought stress (50% of full irrigation) inducing intermediate accumulation (0.120 ± 0.012 μmol/g) between the control and severe drought stress imposed by PEG (20% PEG 6000 solution). Annual treatment effects (Tables 4, S5) showed consistent proline elevation under water deficit across both years, though with varying magnitudes.

Proline serves as a primary osmolyte under drought stress, functioning in cellular protection and osmotic regulation as established in crop species. Sun et al.^67^ demonstrated similar proline accumulation patterns in strawberry under progressive drought stress, with responses correlating to stress severity and duration. Radhi and Abdul-Hasan^68^ further confirmed that exogenous proline application mitigated PEG-induced drought stress in strawberries by enhancing endogenous proline biosynthesis. The complex role of proline in stabilizing cellular structures and supporting stress tolerance mechanisms has been well-documented by Chaitanya et al.^69^, supporting the observed cultivar-specific osmotic adjustment strategies in raspberry.

##### Abscisic acid (ABA) content

Analysis of variance demonstrated significant effects of year (P < 0.001), cultivar (*P* < 0.05), and treatment (*P* < 0.001) on ABA content, with significant year × treatment (*P* < 0.01) and cultivar × treatment (*P* < 0.001) interactions (Table 1). ABA levels were slightly higher in 2024 (94.53 ± 1.45 ng/g) compared to 2022 (92.79 ± 1.23 ng/g), while ‘Jade’ showed marginally higher concentrations (94.15 ± 1.34 ng/g) than ‘Diamond Jubilee’ (93.17 ± 1.12 ng/g) across treatments (Table 2).

Water deficit induced significant ABA accumulation, increasing from 90.11 ± 1.01 ng/g under optimal irrigation to 97.21 ± 1.45 ng/g under PEG treatment (Table 2). Cultivar-specific responses revealed differential ABA signaling patterns, with ‘Diamond Jubilee’ showing 11.9% increase from 87.93 ± 1.12 to 98.41 ± 1.67 ng/g, while ‘Jade’ exhibited a more modest 4.0% elevation from 92.30 ± 1.34 to 96.01 ± 1.89 ng/g under water stress (Table 3). The temporal analysis (Table 4, S5) confirmed year-dependent responses, with consistent ABA elevation patterns maintained across both experimental years but with varying magnitudes. Three-way interaction analysis (Table S3) revealed complex genotype × environment interactions, particularly evident in the differential ABA responses between years and cultivars.

These results align with Qiu et al.^33^ findings demonstrating that ABA accumulation in raspberry leaves responds to vapor pressure deficits and plays a crucial role in reducing mesophyll conductance even when leaf water status remains relatively stable. The cultivar-specific ABA responses corroborate Perin et al.^70^ observations in strawberries, where ABA-dependent responses to mild drought and salt stress not only alleviate osmotic stress but also enhance fruit quality through regulation of metabolic processes. Furthermore, the differential ABA signaling observed supports Villalobos-González et al.^71^ findings in grapes, where ABA engagement in drought-induced secondary metabolism alterations promotes anthocyanin and flavonol biosynthesis during ripening, suggesting similar regulatory mechanisms may operate in raspberry stress adaptation.

### Enzymatic activity

#### Polyphenol oxidase (PPO) activity

Analysis of variance demonstrated highly significant effects of all factors on PPO activity, with year (*P* < 0.001), cultivar (*P* < 0.001), and treatment (*P* < 0.001) showing strong main effects, alongside significant interactions for year × treatment, cultivar × treatment, and the three-way interaction (all *P* < 0.001, Table 1). PPO activity was substantially higher in 2024 (207.75 ± 12.32 U/g/min) compared to 2022 (120.25 ± 8.45 U/g/min), while ‘Diamond Jubilee’ exhibited higher baseline activity (172.58 ± 9.87 U/g/min) than ‘Jade’ (155.42 ± 8.23 U/g/min) across treatments (Table 2).

Water deficit treatment significantly reduced PPO activity from 200.17 ± 11.23 U/g/min under optimal irrigation to 127.83 ± 7.89 U/g/min under PEG stress (Table 2). Cultivar-specific responses revealed distinct patterns: ‘Diamond Jubilee’ showed a 44.8% reduction from 222.34 ± 15.23 to 122.82 ± 9.87 U/g/min, while ‘Jade’ experienced a more moderate 25.4% decrease from 178.00 ± 12.45 to 132.84 ± 8.67 U/g/min under water stress (Table 3). The temporal dynamics analysis (Table S3) revealed dramatic year-dependent responses, with 2024 showing particularly high baseline activities in ‘Diamond Jubilee’ (336 ± 15.23 U/g/min under control conditions) followed by substantial stress-induced reductions (55.4% decrease to 150 ± 9.87 U/g/min under PEG). The supplementary annual analysis confirmed significant cultivar × treatment interactions in both years (P < 0.001, Tables S1, S2), indicating consistent genotypic differences in PPO stress responses.

PPO represents a critical component in plant stress defense mechanisms, catalyzing the oxidation of phenolic compounds to quinones during stress responses^72^. The observed stress-induced reduction in PPO activity aligns with Thipyapong et al.^73^ findings, where PPO suppression was associated with improved water relations and reduced photoinhibition under stress conditions. This enzymatic down-regulation may represent an adaptive mechanism to prevent excessive photooxidative damage during drought stress, as demonstrated by Thipyapong et al.^74^ described PPO-mediated pathways implicated in stress tolerance, depending on context, PPO activity can either contribute to defense via phenolic oxidation or, when down-regulated, be associated with reduced photodamage.

#### Peroxidase (POD) activity

**Fig. 3:**
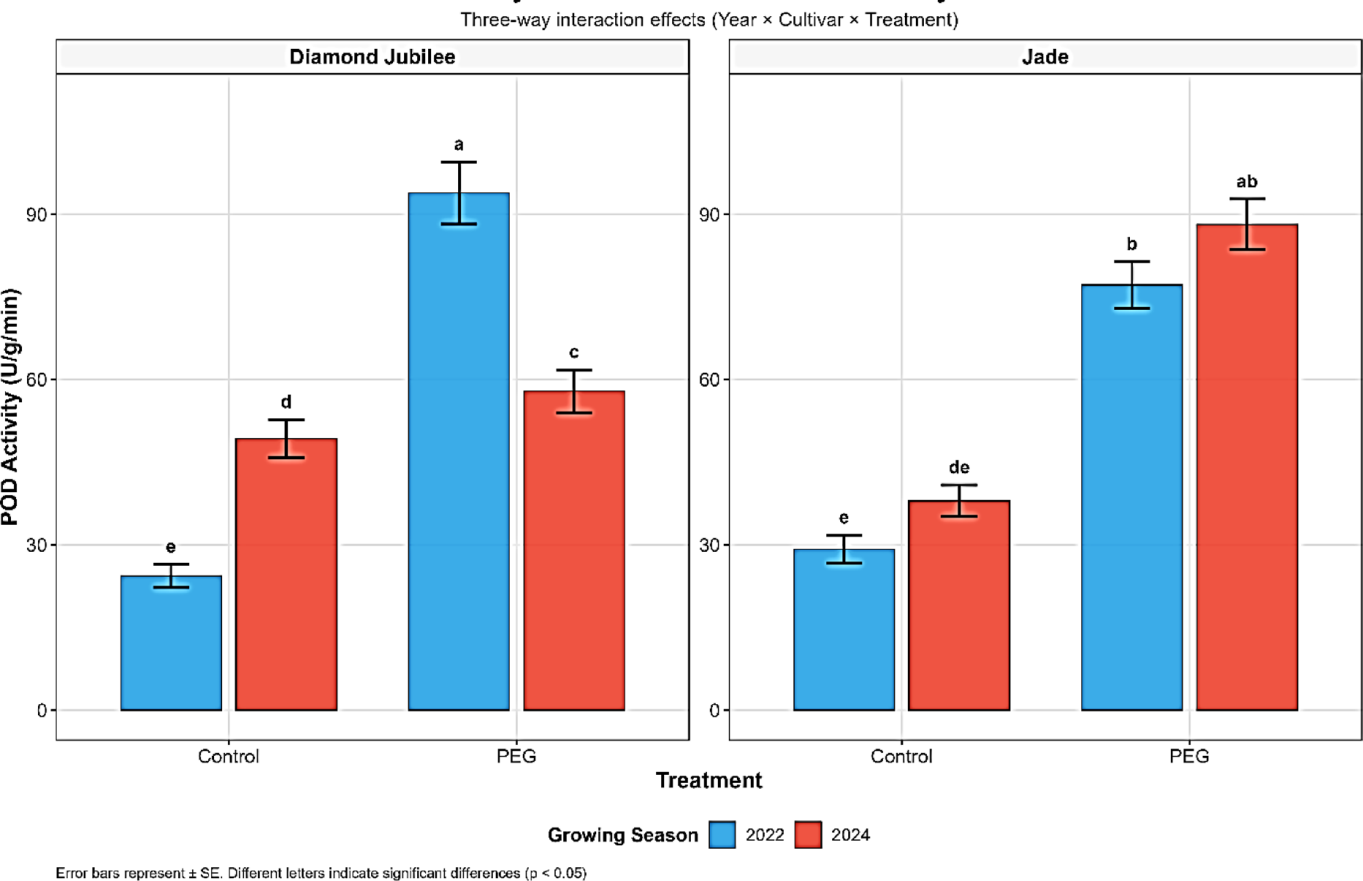
Three-way interaction effects (Year × Cultivar × Treatment) on peroxidase (POD) activity (U g^−1^ min^−1^) in raspberry cultivars ‘Diamond Jubilee’ and ‘Jade’ under control and PEG-induced drought stress conditions across two growing seasons (2022 and 2024).

Peroxidase (POD) activity showed cultivar-specific responses to drought stress (Fig. 3). In 2022, ‘Diamond Jubilee’ exhibited a clear, dose–response POD activity enhancement, from 24.40 U/g/min with full irrigation to 62.60 U/g/min (a 156.6% enhancement) with 50% irrigation, and up to 93.90 U/g/min (a 284.8% enhancement) with PEG-induced stress, indicating a strong and proportionate antioxidant defense (Fig. 3). On the other hand, ‘Jade’ showed a biphasic response: POD activity decreased by 65.4% under moderate water stress (from 29.20 to 10.10 U/g/min), but increased abruptly by 164.4% under PEG treatment (to 77.20 U/g/min) (Table 3, Fig. 3). These non-linear responses might represent genotype-dependent thresholds for enzymatic defense induction, as also shown by Zhang et al.^75^, who reported significant POD upregulation in Rubus species in water stress. In 2024, treatment with PEG induced a moderate 17.4% increase in ‘Diamond Jubilee’ and a significantly larger 132.1% increase in ‘Jade’, once again consistent with evidence supporting the role of POD as a functional stress-reactive enzyme (Fig. 3). Our findings accord with those of Morariu et al.^76^, who indicated POD activity in raspberry as an essential component of a water and light stress reaction.

#### Leaf dry matter content

Water deficit significantly increased LDMC across both years (P < 0.001), with ‘Diamond Jubilee’ showing greater increases (63.2% from 27.19 to 44.39%) than ‘Jade’ (51.6% from 26.72 to 40.51%) under PEG treatment (Table 3). Higher LDMC values coincided with reduced stomatal conductance and transpiration rates, reflecting structural adaptations that complement stomatal responses during drought stress^77^. Plants with enhanced structural investment through elevated LDMC maintain water retention when stomatal regulation alone is insufficient, as demonstrated in drought tolerance studies where LDMC positively correlates with gas exchange efficiency under water limitation^78,79^. The greater LDMC response in ‘Diamond Jubilee’ potentially compensates for its reduced water retention capacity, representing an integrated physiological strategy combining structural and gas exchange modifications for drought adaptation.

### Gas exchange parameters

#### Leaf transpiration rate (ATranspLeaf)

Analysis of variance revealed significant cultivar effects (*P* < 0.05) and highly significant drought stress effects (*P* < 0.001) on leaf transpiration rate, with significant cultivar × drought interactions (*P* < 0.01, Table 5). ‘Diamond Jubilee’ exhibited significantly higher baseline transpiration rates (1.84 ± 0.09 mmol m^−2^ s^−1^) compared to ‘Jade’ (1.29 ± 0.13 mmol m^−2^ s^−1^), indicating intrinsic physiological differences between cultivars.

**Table 5:** Analysis of variance (ANOVA) and mean comparisons for physiological, morphological, and biochemical traits of raspberry cultivars under different drought stress treatments in 2022.

Source of Variation	Df	Leaf transpiration rate (mmol m^−2^ s^−1^)	Intercellular CO₂ (μmol mol^−1^)	Stomatal conductance (mmol m^−2^ s^−1^)	Leaf assimilation rate (μmol m^−2^ s^−1^)	Leaf area (cm^2^)	Petiole length (cm)	Leaf succinic acid (mg g^−1^ DW)
Cultivar	1	1.383*	4439.961 ns	1817.643*	45.506***	386.976**	6.009***	42.524**
Drought stress	2	19.815***	220,416***	42,401***	67.689***	203.027**	0.735*	43.050 **
Cultivar × Drought stress	2	2.310**	7176.773 ns	3609.976**	15.962**	272.272**	0.357 ns	12.177*

Efects	Treatment	Leaf transpiration rate (mmol m^−2^ s^−1^)	Intercellular CO₂ (μmol mol^−1^)	Stomatal conductance (mmol m^−2^ s^−1^)	Leaf assimilation rate (μmol m^−2^ s^−1^)	Leaf area (cm^2^)	Petiole length (cm)	Leaf succinic acid (mg g^−1^ DW)
Cultivar	Diamond Jubilee	1.84 ± 0.09 a	558.1 ± 21.4a	75.0 ± 9.6 a	2.20 ± 0.48 a	36.2 ± 1.9 a	4.74 ± 0.23 a	12.1 ± 0.6 a
	Jade	1.29 ± 0.13 b	526.7 ± 48.6a	54.9 ± 11.1 b	− 0.98 ± 0.52 b	26.9 ± 1.1 b	3.59 ± 0.17 b	9.06 ± 0.53 b
Drought stress	100%	3.66 ± 0.20 a	336.9 ± 5.8 c	161.7 ± 7.4 a	4.18 ± 0.40 a	33.1 ± 2.8 a	4.52 ± 0.28 a	13.6 ± 0.8 a
	50%	0.67 ± 0.21 b	574.2 ± 32.5 b	23.6 ± 8.3 b	0.16 ± 0.32 b	36.4 ± 2.1 a	3.82 ± 0.19 b	9.69 ± 0.66 b
	PEG	0.37 ± 0.12 b	716.2 ± 48.1 a	9.61 ± 2.0 b	− 2.50 ± 0.36 c	25.1 ± 2.1 b	4.17 ± 0.13 ab	8.49 ± 0.36 b
Cultivar × Drought stress	100% × Diamond Jubilee	4.65 ± 0.02 a	313.4 ± 0.2a	199.9 ± 0.8 a	6.91 ± 0.01 a	45.5 ± 5.0 a	5.13 ± 0.28	16.5 ± 1.5 a
	100% × Jade	2.66 ± 0.43 b	360.4 ± 11.4a	123.6 ± 14.9 b	1.45 ± 0.80 b	20.8 ± 0.6 c	3.90 ± 0.29	10.7 ± 0.8 b
	50% × Diamond Jubilee	0.57 ± 0.01 c	616.1 ± 3.6a	16.6 ± 0.2 c	2.48 ± 0.01 b	36.2 ± 2.8 ab	4.13 ± 0.23	9.73 ± 0.82 bc
	50% × Jade	0.76 ± 0.41 c	532.3 ± 61.5a	30.7 ± 16.8 c	− 2.16 ± 0.64 c	36.5 ± 2.4 ab	3.50 ± 0.15	9.65 ± 0.49 bc
	PEG × Diamond Jubilee	0.31 ± 0.07 c	744.9 ± 23.3a	8.67 ± 2.0 c	− 2.77 ± 0.07 c	26.7 ± 2.0 bc	4.97 ± 0.18	10.2 ± 0.4 bc
	PEG × Jade	0.44 ± 0.16 c	687.5 ± 72.8a	10.6 ± 2.0 c	− 2.22 ± 0.64 c	23.4 ± 2.2 bc	3.37 ± 0.09	6.80 ± 0.36 c

Values represent means ± SE. Different letters within each column indicate significant differences among cultivars, drought treatments, and their interactions at *P* ≤ 0.05 according to Tukey’s HSD test. Results are presented only for parameters showing significant effects in 2022.

Progressive drought stress induced severe reductions in transpiration rates across treatments, from 3.66 ± 0.20 mmol m^−2^ s^−1^ under optimal irrigation (100%) to 0.67 ± 0.21 mmol m^−2^ s^−1^ under moderate stress (50%) and 0.37 ± 0.12 mmol m^−2^ s^−1^ under severe PEG treatment (Table 5). Cultivar-specific responses demonstrated differential stress sensitivity: under optimal conditions, ‘Diamond Jubilee’ maintained superior transpiration rates (4.65 ± 0.02 mmol m^−2^ s^−1^) compared to ‘Jade’ (2.66 ± 0.43 mmol m^−2^ s^−1^). However, ‘Diamond Jubilee’ experienced more severe stress-induced reductions, declining by 87.7% under moderate stress (0.57 ± 0.01 mmol m^−2^ s^−1^) and 93.3% under PEG treatment (0.31 ± 0.07 mmol m^−2^ s^−1^), while ‘Jade’ showed relatively smaller decreases of 71.4% (0.76 ± 0.41 mmol m^−2^ s^−1^) and 83.5% (0.44 ± 0.16 mmol m^−2^ s^−1^), respectively.

These findings align with Morales et al.^35^ observations of rapid stomatal closure as the primary defense mechanism in Rubus species under water limitation. The greater transpiration decline in ‘Diamond Jubilee’ suggests this cultivar employs a more conservative water-use strategy during stress conditions compared to the relatively stress-tolerant response exhibited by ‘Jade’.

#### Intercellular CO_2_ concentration (InCO_2_Leaf)

Analysis of variance showed non-significant cultivar effects but highly significant drought stress effects (*P* < 0.001) on intercellular CO₂ concentration, with non-significant cultivar × drought interactions (Table 5). Both cultivars maintained similar CO₂ levels (‘Diamond Jubilee’: 558.1 ± 21.4 μmol mol^−1^; ‘Jade’: 526.7 ± 48.6 μmol mol^−1^). Drought stress induced progressive CO₂ accumulation, increasing from 336.9 ± 5.8 μmol mol^−1^ under optimal irrigation to 574.2 ± 32.5 μmol mol^−1^ under moderate stress and 716.2 ± 48.1 μmol mol^−1^ under PEG treatment (Table 5). Cultivar-specific responses showed ‘Diamond Jubilee’ accumulated higher CO₂ levels under severe stress (744.9 ± 23.3 μmol mol^−1^) compared to ‘Jade’ (687.5 ± 72.8 μmol mol^−1^), despite similar responses under moderate stress conditions. The stress-induced CO₂ accumulation indicates non-stomatal limitations rather than diffusion constraints as the primary cause of photosynthetic inhibition, likely reflecting reduced Calvin cycle activity under water deficit conditions. The greater CO₂ accumulation in ‘Diamond Jubilee’ under severe stress suggests enhanced metabolic limitations compared to ‘Jade’.

#### Stomatal conductance (GSWLeaf)

Stomatal conductance (GSWLeaf) was significantly influenced by cultivar (*p* < 0.05), drought treatment (*p* < 0.001), and their interaction (p < 0.01) (Table 5). Among cultivars, ‘Diamond Jubilee’ demonstrated significantly higher stomatal conductance (75.0 ± 9.6 mmol m^−2^ s^−1^) compared to ‘Jade’ (54.9 ± 11.1 mmol m^−2^ s^−1^).Drought treatments induced dramatic reductions in stomatal conductance across both cultivars (Table 5). Under optimal irrigation (100% treatment), stomatal conductance reached 161.7 ± 7.4 mmol m^−2^ s^−1^, which was significantly reduced to 23.6 ± 8.3 mmol m^−2^ s^−1^ under moderate stress (50% treatment) and further declined to 9.61 ± 2.0 mmol m^−2^ s^−1^ under severe PEG-induced stress.The cultivar × drought interaction revealed distinct responses between cultivars (Table 5). Under non-stress conditions, ‘Diamond Jubilee’ exhibited the highest stomatal conductance (199.9 ± 0.8 mmol m^−2^ s^−1^), significantly exceeding ‘Jade’ (123.6 ± 14.9 mmol m^−2^ s^−1^). Both cultivars showed severe reductions under stress treatments, with PEG treatment causing the most pronounced stomatal closure in both ‘Diamond Jubilee’ (95.7% reduction to 8.67 ± 2.0 mmol m^−2^ s^−1^) and ‘Jade’ (91.4% reduction to 10.6 ± 2.0 mmol m^−2^ s^−1^). These findings align with previous research by Qiu et al.^33^ and Lepaja et al. (2020), confirming that stomatal closure serves as a primary water conservation mechanism during drought stress in berry crops.

#### Net assimilation rate (AssimLeaf)

The net CO₂ assimilation rate (AssimLeaf) was significantly influenced by cultivar (*p* < 0.001), drought treatment (*p* < 0.001), and their interaction (*p* < 0.01). Analysis of variance revealed that cultivar differences, drought stress levels, and their interactions all played crucial roles in determining photosynthetic performance, consistent with findings by Bhusal et al.^80^ who demonstrated that plant responses to drought are consistently related to cultivar differences and stress intensity. Under optimal irrigation conditions (100% treatment), the overall assimilation rate reached 4.18 ± 0.40 μmol m^−2^ s^−1^, which progressively declined to 0.16 ± 0.32 μmol m^−2^ s^−1^ under moderate stress (50% treatment) and became severely negative at − 2.50 ± 0.36 μmol m^−2^ s^−1^ under PEG-induced stress. Between cultivars, ‘Diamond Jubilee’ demonstrated significantly higher photosynthetic capacity (2.20 ± 0.48 μmol m^−2^ s^−1^) compared to ‘Jade’ (− 0.98 ± 0.52 μmol m^−2^ s^−1^), indicating superior photosynthetic performance under the experimental conditions.

The cultivar × drought interaction revealed distinct photosynthetic responses. Under full irrigation, ‘Diamond Jubilee’ exhibited substantially higher assimilation rates (6.91 ± 0.01 μmol m^−2^ s^−1^) than ‘Jade’ (1.45 ± 0.80 μmol m^−2^ s^−1^), demonstrating superior photosynthetic capacity under optimal conditions, as reported by Yang et al.^43,44^ for drought-resistant cultivars under different water conditions.

However, under severe PEG-induced stress, both cultivars exhibited negative assimilation rates, with ‘Diamond Jubilee’ showing − 2.77 ± 0.07 μmol m^−2^ s^−1^ and ‘Jade’ − 2.22 ± 0.64 μmol m^−2^ s^−1^. These negative values indicate that respiratory CO₂ production exceeded photosynthetic CO₂ absorption, representing a critical tipping point where plants shift to negative carbon balance^81^. Interestingly, ‘Diamond Jubilee’, despite its superior performance under optimal conditions, showed more pronounced negative assimilation under extreme stress, suggesting greater vulnerability to severe water deficit compared to ‘Jade’. This paradoxical response highlights the complex relationships between photosynthetic capacity, genetic sensitivity, and stress adaptation mechanisms^82,83^.

#### Leaf morphological traits

Analysis of variance conducted in 2022 revealed significant cultivar effects on leaf area (*P* < 0.01) and petiole length (*P* < 0.001), with drought stress also exerting significant effects on both traits (*P* < 0.01 and *P* < 0.05, respectively; Table 5). A significant cultivar × drought interaction was detected for leaf area (*P* < 0.01) but not for petiole length. Across treatments, *Diamond Jubilee* consistently exhibited larger leaf area (36.2 ± 1.9 cm^2^) than *Jade* (26.9 ± 1.1 cm^2^). Drought reduced mean leaf area from 33.1 ± 2.8 cm^2^ under full irrigation to 25.1 ± 2.1 cm^2^ under PEG, while moderate stress produced intermediate values (36.4 ± 2.1 cm^2^). Such reductions are a common morphological adaptation that conserves water^84^, although they can directly constrain photosynthetic capacity and fruit production because leaf area and expansion are key determinants of carbon gain and dry-matter accumulation^85,86^.

The cultivar × drought interaction revealed contrasting strategies: under optimal conditions *Diamond Jubilee* produced substantially larger leaves (45.5 ± 5.0 cm^2^) than *Jade* (20.8 ± 0.6 cm^2^). Under moderate stress, however, *Jade* increased leaf area to 36.5 ± 2.4 cm^2^ while *Diamond Jubilee* decreased to 36.2 ± 2.8 cm^2^; under severe PEG stress both cultivars showed comparable strong reductions. These genotype-specific responses reflect different drought-tolerance strategies and emphasize the trade-off between water conservation and light capture^43,44^.

Petiole length also favored *Diamond Jubilee* (4.74 ± 0.23 cm) over *Jade* (3.59 ± 0.17 cm), with drought reducing values from 4.52 ± 0.28 cm under optimal irrigation to 4.17 ± 0.13 cm under PEG. Such morphological adjustments likely contribute to stress avoidance^85^, but they simultaneously reduce the photosynthetic surface available for carbohydrate production, with possible negative consequences for fruit development and quality^87^.

Overall, the observed morphological adaptations indicate a clear trade-off between drought survival and productivity: maintenance of larger leaf area may support productivity under mild stress, whereas severe drought causes reductions that threaten yield. These results align with previous reports in *Rubus* describing leaf structural modifications as effective drought-avoidance strategies (Percival, 1998; Yang et al., 2018).

#### Organic acid composition

The organic acid profiles of ‘Diamond Jubilee’ and ‘Jade’ raspberry cultivars were evaluated exclusively in 2024 and were markedly influenced by PEG-induced drought stress (Table 6), with distinct responses observed between the cultivars and acid types. Organic acids are vital for fruit flavor, quality, and stress response in plants.

**Table 6:** Analysis of variance (ANOVA) and mean comparisons for organic acid composition and antioxidant activity of raspberry cultivars under water stress treatments in 2024.

Source of Variation	Df	Oxalic acid (mg g^−1^ DW)	Citric acid (mg g^−1^ DW)	Malic acid (mg g^−1^ DW)	Ascorbic acid (mg g^−1^ DW)
Cultivar	1	74.81***	728.65***	189.57***	196.56***
Water stress	1	1.69 ns	397.84***	285.02***	5366.56***
Cultivar × Water stress	1	37.78***	92.61***	0.65 ns	196.56***

Effects	Treatment	Oxalic acid (mg g^−1^ DW)	Citric acid (mg g^−1^ DW)	Malic acid (mg g^−1^ DW)	Ascorbic acid (mg g^−1^ DW)
Cultivar	Diamond Jubilee	2.94 ± 0.08 b	5.76 ± 0.21 a	5.19 ± 0.12 a	0.16 ± 0.02 a
	Jade	3.86 ± 0.11 a	3.30 ± 0.14 b	4.22 ± 0.09 b	0.11 ± 0.01 b
Water stress	100%	3.33 ± 0.13 a	3.62 ± 0.16 b	4.11 ± 0.11 b	0.26 ± 0.01 a
	PEG	3.47 ± 0.09 a	5.43 ± 0.18 a	5.30 ± 0.10 a	0.00 ± 0.00 b
Cultivar × Water stress	100% × Diamond Jubilee	2.54 ± 0.05 c	4.41 ± 0.05 b	4.62 ± 0.05 a	0.31 ± 0.01 a
	100% × Jade	4.12 ± 0.14 a	2.83 ± 0.13 d	3.59 ± 0.08 a	0.21 ± 0.00 b
	PEG × Diamond Jubilee	3.33 ± 0.13 b	7.10 ± 0.06 a	5.76 ± 0.08 a	0.00 ± 0.00 c
	PEG × Jade	3.60 ± 0.09 b	3.77 ± 0.11 c	4.84 ± 0.07 a	0.00 ± 0.00 c

Values represent means ± SE. Different letters within each column indicate significant differences among cultivars, water stress treatments, and their interactions at *P* ≤ 0.05 according to Tukey’s HSD test. ***: *P* ≤ 0.001; ns: non-significant. For malic acid, only main effects are presented due to non-significant interaction. DW: dry weight; TE: Trolox equivalent.

Oxalic acid levels differed significantly by cultivar (*p* < 0.001) and cultivar × water stress interaction (*p* < 0.001), though not by water stress alone. ‘Jade’ had higher baseline oxalic acid content (4.12 mg g^−1^ DW) compared to ‘Diamond Jubilee’ (2.54 mg g^−1^ DW) under control conditions. Under stress, ‘Jade’ showed a slight decrease to 3.60 mg g^−1^ DW (12.6% reduction), while ‘Diamond Jubilee’ increased to 3.33 mg g^−1^ DW (31.1% increase). These opposite trends reflect cultivar-specific metabolic adjustments, consistent with genotype-dependent responses reported by Ma et al. (2022).

Citric acid was significantly affected by all factors (*p* < 0.001). ‘Diamond Jubilee’ had higher baseline content (4.41 mg g^−1^ DW) than ‘Jade’ (2.83 mg g^−1^ DW), and both cultivars increased citric acid under PEG-induced drought stress. However, ‘Diamond Jubilee’ showed a sharper rise (61.0% to 7.10 mg g^−1^ DW) than ‘Jade’ (33.2% to 3.77 mg g^−1^ DW). Fuentealba et al. (2024) highlighted the central role of citric acid in raspberry tartness and its sensitivity to environmental cues.

Malic acid content also showed significant changes due to both cultivar (*p* < 0.001) and water stress (*p* < 0.001), with no significant interaction effect. While ‘Diamond Jubilee’ had higher initial levels (4.62 mg g^−1^ DW), both cultivars increased similarly under drought (24.7% and 34.8%, respectively), reaching 5.76 and 4.84 mg g^−1^ DW. As suggested by Zheng et al. (2019), such increases may serve in osmotic regulation and reflect enhanced carbon metabolism under stress.

Ascorbic acid (vitamin C) levels were significantly influenced by all factors (*p* < 0.001). Both cultivars contained measurable amounts under control conditions (‘Diamond Jubilee’: 0.31 mg g^−1^ DW; ‘Jade’: 0.21 mg g^−1^ DW), yet PEG stress in 2024 completely depleted ascorbic acid in both cultivars. This is consistent with recent findings showing that drought-induced oxidative stress significantly depletes ascorbic acid levels in plant tissues, as reported in soybean leaves^88^. The rapid utilization of ascorbic acid highlights its critical role in antioxidant defense. These findings underscore the dynamic nature of organic acid metabolism under drought stress and the importance of genotype in shaping these responses. Famiani and Walker (2009) noted the complexity of organic acid metabolism in Rubus species, highlighting that the greatest impact in metabolic adjustments often occurs through enzymatic regulation. Similarly, Ipek (2019) emphasized that such metabolic shifts can affect nutrient uptake and overall plant physiology. Ultimately, the changes in organic acids observed in 2024 will have significant implications for fruit quality, flavor, and stress tolerance.

#### PCA analysis

**Fig. 4:**
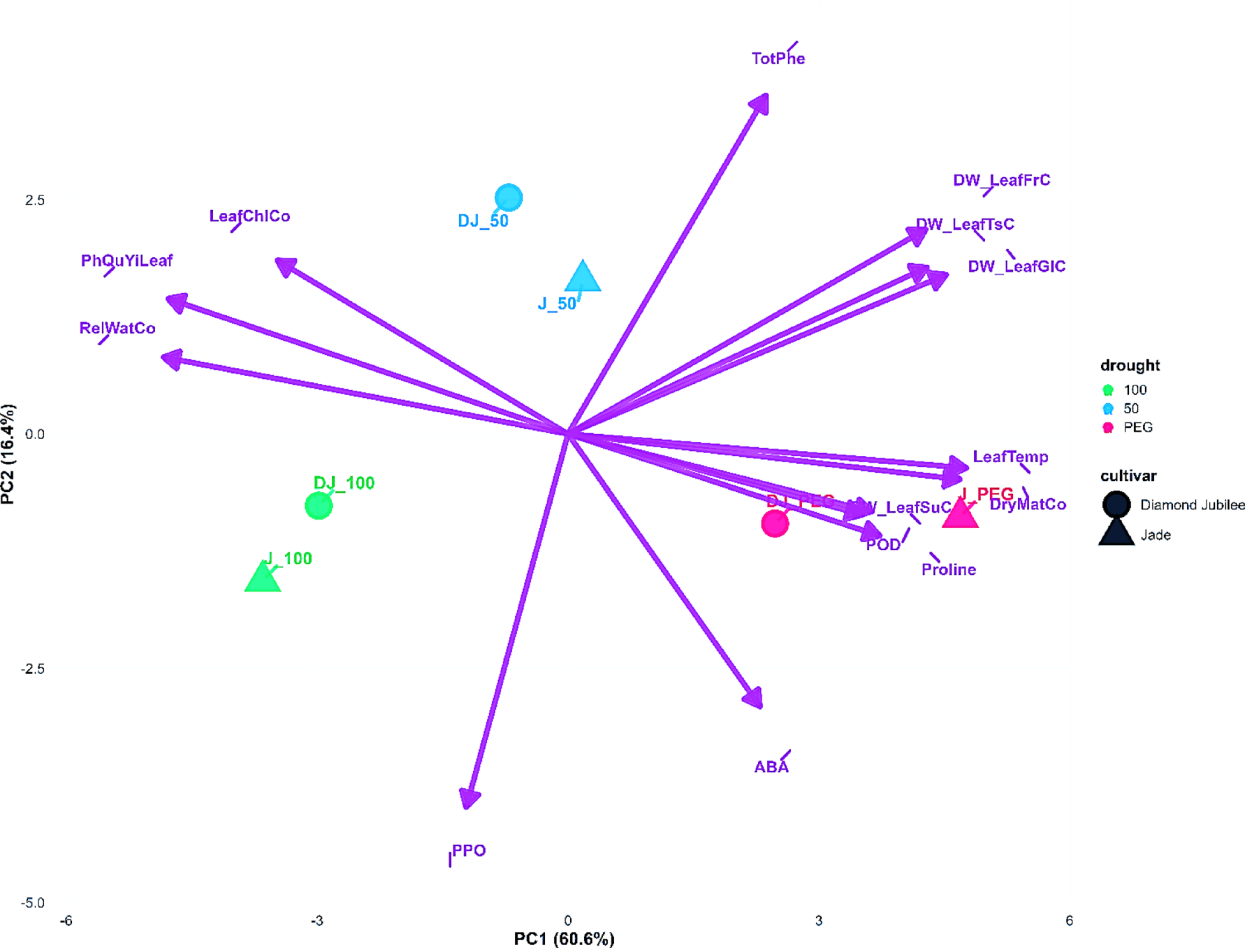
Principal component analysis (PCA) biplot of physiological traits in raspberry cultivars under different water stress treatments (100% field capacity, 50% field capacity, and PEG-induced stress) in 2022.

The PCA biplot analysis revealed distinct physiological and biochemical response patterns in Diamond Jubilee and Jade raspberry cultivars under different irrigation treatments. For 2022 (Fig. 4), the first two principal components explained 77.1% of total variance (PC1: 60.6%, PC2: 16.5%). Clear separation was observed among water treatments (100% irrigation, 50% irrigation, and PEG treatment), with the 50% irrigation treatment positioned intermediately between the control and stress conditions. Diamond Jubilee and Jade cultivars occupied different positions under identical treatments, indicating genotype-specific stress responses.

Water-related parameters including RelWatCo (relative water content) and LeafTemp (leaf temperature) showed distinct positioning in the biplot, with RelWatCo showing negative correlation to PC1. Photosynthetic parameters including PhQyYiLeaf (quantum yield), LeafChiCo (leaf chlorophyll content), and carbohydrate parameters (DW_LeafSuC, DW_LeafTsC, DW_LeafFrC, DW_LeafGIC) were distributed across different regions of the biplot, with most showing negative correlations to PC1, confirming their coordinated response under water deficit conditions. Enzyme activity parameters PPO (polyphenol oxidase) and POD (peroxidase) were positioned in different quadrants, indicating their distinct roles in plant stress responses. TotPhe (total phenolic compounds) showed specific positioning, suggesting its involvement in stress defense mechanisms. ABA was positioned in the negative PC2 region, representing its role in stress signaling, while Proline showed distinct positioning indicating its specific function in osmotic adjustment under drought conditions.

**Fig. 5:**
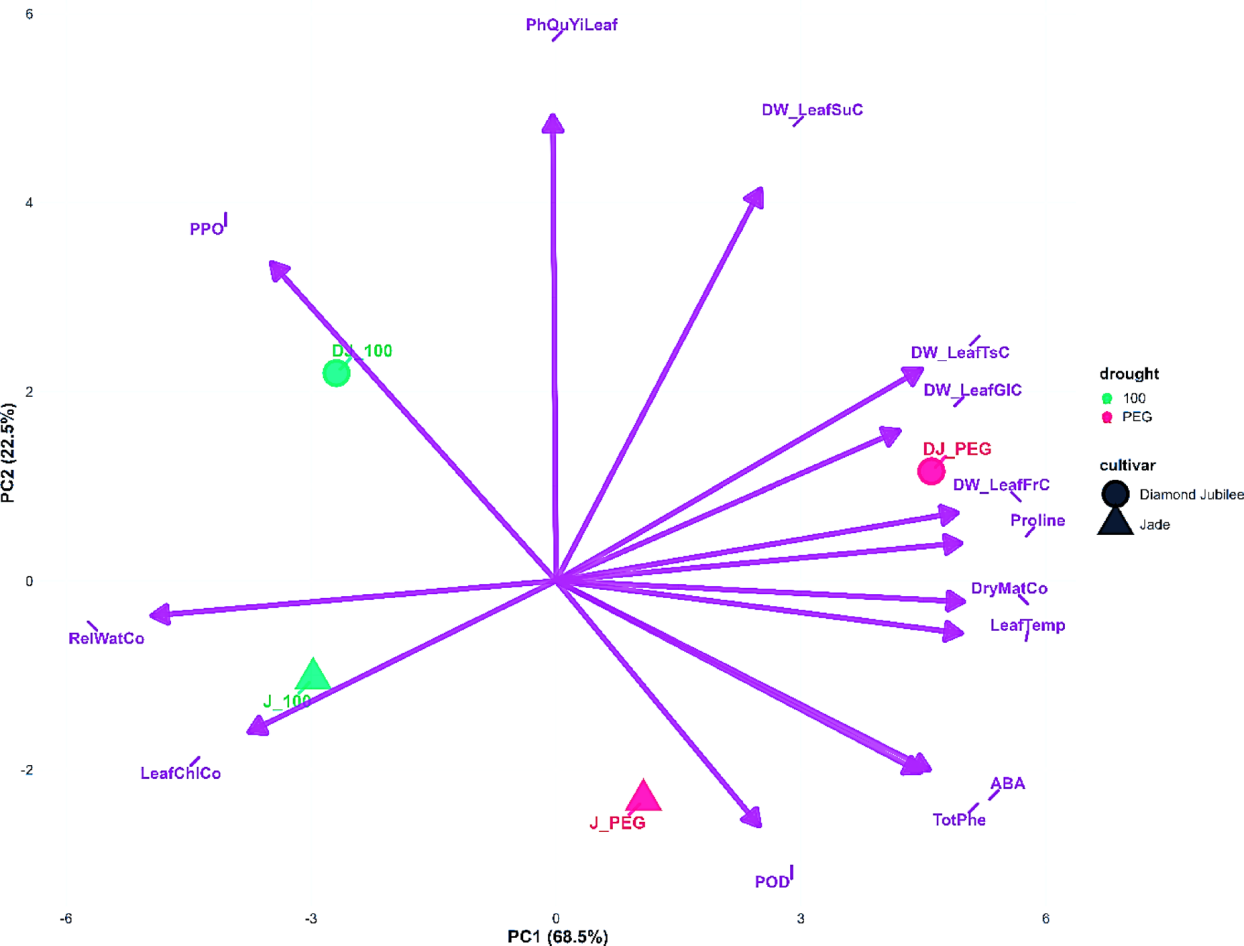
Principal component analysis (PCA) biplot of physiological traits in raspberry cultivars under different water stress treatments (100% field capacity and PEG-induced stress) in 2024.

The 2024 PCA biplot (Fig. 5) showed increased explained variance (90.2%) by the first two PCs (PC1: 68.5%, PC2: 21.7%), indicating more coordinated physiological responses following prolonged treatment exposure. The separation between treatments and cultivars became more pronounced, with greater distances observed between the 100% irrigation control and PEG stress treatments, reflecting the cumulative effects of prolonged water stress.

Notable changes in parameter positioning occurred between the two years: PhQyYiLeaf showed altered correlation patterns, suggesting modified photosynthetic responses under prolonged stress. The enzyme parameters PPO and POD maintained significant presence in the biplot, indicating sustained enzymatic activity adjustments. ABA positioning relative to other stress-related parameters suggested evolving roles in the transition from immediate stress signaling to long-term physiological adaptation mechanisms. Carbohydrate metabolism parameters (sucrose, glucose, fructose, and total sugar) underwent repositioning in the biplot space, indicating altered sugar partitioning and metabolic adjustments under continuous water limitation conditions.

## Conclusion

This two-year study comprehensively evaluated the physiological and biochemical responses of two raspberry cultivars, ‘Diamond Jubilee’ and ‘Jade’, under both soil water deficit and PEG-induced osmotic stress. The results revealed genotype-specific strategies for coping with drought. ‘Jade’ maintained higher relative water content, chlorophyll levels, and photosynthetic efficiency under stress conditions, particularly in the second year, indicating superior physiological resilience. In contrast, ‘Diamond Jubilee’ displayed a more robust biochemical response, characterized by significant accumulation of proline, phenolics, and antioxidant enzyme activities (especially peroxidase), particularly in the second season—suggesting enhanced osmotic and oxidative defense mechanisms. Soluble sugar accumulation (notably glucose and fructose) increased markedly under both types of stress in both years, with greater magnitudes in 2022. This likely reflects an osmotic adjustment mechanism that was partially retained in 2024, indicative of stress memory effects. Organic acids such as citric and malic acids increased under stress, with cultivar-dependent trends, while ascorbic acid was depleted entirely in both cultivars, underlining the severity of oxidative stress. PEG treatments generally triggered stronger responses than moderate water reduction, highlighting the intensity of osmotic stress. Principal component analysis clearly separated cultivar responses and treatment effects, with consistent clustering across years. Overall, ‘Jade’ appears more physiologically stable, while ‘Diamond Jubilee’ exhibits stronger metabolic plasticity. The most informative traits for rapid drought screening included RWC, proline content, SPAD chlorophyll, and peroxidase activity. These findings provide a valuable basis for selecting drought-resilient raspberry genotypes and designing stress-mitigation strategies in breeding programs.

## Supplementary Information

Below is the link to the electronic supplementary material.

Supplementary Material 1

## Data Availability

All data generated or analyzed during this study are included in this article. All materials are available through the corresponding authors upon reasonable request
