# Supplementary material for: A comprehensive analysis of the physiological and biochemical responses of raspberry cultivars to water stress
Source: Sci Rep. 2025 Nov 24;15:43707. doi: 10.1038/s41598-025-27434-7 (PMC12700926; doi:10.1038/s41598-025-27434-7)
Supplement: Supplementary file 1 — Supplementary Material 1 [file 41598_2025_27434_MOESM1_ESM.docx]

**SUPPLEMENTARY TABLES**

**Table S1. Analysis of variance (ANOVA) for physiological, biochemical, and antioxidant traits in raspberry cultivars under different treatments (year 1)**

| **Source** | **Cultivar** | | **Treatment** | | **Cultivar × Treatment** |
| --- | --- | --- | --- | --- | --- |
| DF | 1 | 2 | | 2 | |
| Leaf Temp (°C) | 0.20 ^ns^ | 4.76 ^***^ | | 0.16 ^ns^ | |
| RWC (%) | 0.03 ns | 2228.06 *** | | 154.26 *** | |
| Chlorophyll (µmol/m²) | 16.18 ns | 51.09 *** | | 14.46 * | |
| Photosynthetic Quantum Yield of Leaf | 0.00 * | 0.00 *** | | 0.00 ns | |
| Dried Leaf Sucrose Content (%) | 2.86 *** | 0.94 ** | | 0.17 ns | |
| Dried Leaf Glucose Content (%) | 2.92 ns | 14.20 *** | | 1.30 ns | |
| Dried Leaf Fructose Content (%) | 0.55 ns | 5.91 *** | | 0.20 ns | |
| Dried Leaf Total Sugar (%) | 11.31 * | 44.34 *** | | 3.04 ns | |
| Total Phenol (mg GAE/100g) | 0.30 ns | 4036.78 ns | | 2310.78 ns | |
| Leaf Dry Matter Content (%) | 3.14 ns | 468.36 ** | | 17.44 ns | |
| Proline (μmol/g) | 1.70 *** | 2.14 *** | | 2.10 *** | |
| ABA (ng/g) | 119.61 *** | 109.52 *** | | 102.52 *** | |
| POD (U/g/min) | 2073.68 *** | 5963.41 *** | | 1256.80 *** | |
| PPO (U/g/min) | 2403.56 *** | 3531.06 *** | | 775.06 *** | |

Significance levels: ***P < 0.001, **P < 0.01, *P < 0.05, ns = not significant.*

**Table S2. Analysis of variance (ANOVA) for physiological, biochemical, and antioxidant traits in raspberry cultivars under different treatments (year 2)**

| Source | Cultivar | | Treatment | | Cultivar × Treatment |
| --- | --- | --- | --- | --- | --- |
| DF | 1 | 1 | | 1 | |
| Leaf Temp (°C) | 0.36 ns | 8.33 *** | | 0.96 ns | |
| Chlorophyll (µmol/m²) | 14.74 *** | 21.07 *** | | 4.94 ** | |
| RWC (%) | 827.18 ** | 4640.15 *** | | 938.99 ** | |
| Photosynthetic Quantum Yield of Leaf | 0.03 *** | 0.00 * | | 0.00 ns | |
| Dried Leaf Sucrose Content (%) | 2.67 *** | 0.17 ns | | 0.02 ns | |
| Dried Leaf Glucose Content (%) | 0.63 * | 0.77 * | | 1.06 ** | |
| Dried Leaf Fructose Content (%) | 1.03 * | 3.90 ** | | 0.10 ns | |
| Dried Leaf Total Sugar (%) | 11.57 ** | 10.73 ** | | 1.46 ns | |
| Total Phenol (mg GAE/100g) | 2258.94 ns | 106093.99 *** | | 22190.91 ** | |
| Leaf Dry Matter Content (%) | 56.07 * | 671.40 *** | | 34.41 * | |
| Proline (μmol/g) | 0.09 *** | 0.50 *** | | 0.09 *** | |
| ABA (ng/g) | 2.52 ns | 207.83 *** | | 19.87 ** | |
| POD (U/g/min) | 270.75 * | 2593.08 *** | | 1297.92 *** | |
| PPO (U/g/min) | 14630.08 *** | 46252.08 *** | | 11470.08 *** | |

Significance levels: ***P < 0.001, **P < 0.01, *P < 0.05, ns = not significant.*

**Table S3. Complex three-way interactions (year × cultivar × treatment) effects on critical stress response indicators in raspberry plants during two growing seasons (2022-2024)**

| Year | | Cultivar | Treatment | | Water Relations | |  | | Osmotic Adjustment | | Enzymatic Defense | |  | |
| --- | --- | --- | --- | --- | --- | --- | --- | --- | --- | --- | --- | --- | --- | --- |
|  | |  |  | | RWC (%) | | Leaf Temp (°C) | | Proline (μmol/g) | | POD (U/g/min) | | PPO (U/g/min) | |
| 2022 | | Diamond Jubilee | 100% | | 83.50±2.34 a | | 20.8±0.45 f | | 0.037±0.004 e | | 24.4±2.12 e | | 109±8.45 d | |
|  | |  | PEG | | 56.03±3.12 c | | 22.23±0.52 e | | 0.114±0.012 d | | 93.9±5.67 a | | 96±7.23 d | |
|  | | Jade | 100% | | 95.00±1.89 a | | 20.23±0.41 f | | 0.021±0.003 ef | | 29.2±2.56 e | | 152±9.87 c | |
|  | |  | PEG | | 48.15±2.89 d | | 22.3±0.48 e | | 0.209±0.009 c | | 77.2±4.23 b | | 124±8.67 cd | |
| 2024 | Diamond Jubilee | | | 100% | | 89.40±2.12 a | | 28.85±0.67 b | | 0.018±0.002 f | | 49.3±3.45 d | | 336±15.23 a |
|  |  | | | PEG | | 32.38±3.45 e | | 31.08±0.78 a | | 0.598±0.045a | | 57.9±3.89 c | | 150±9.87 c |
|  | Jade | | | 100% | | 88.32±1.98 a | | 29.07±0.71 b | | 0.015±0.002 f | | 38.0±2.87 de | | 204±12.45 b |
|  |  | | | PEG | | 66.68±4.12 b | | 30.17±0.69 ab | | 0.250±0.023 b | | 88.2±4.56 ab | | 142±8.23 c |

*Values represent means ± SE. Different letters indicate significant differences across all treatment combinations (P ≤ 0.05, Tukey's HSD test). Only parameters showing significant three-way interactions (P < 0.001) are presented.*

**Table S4. Annual analysis of carbohydrate metabolism and osmotic adjustment compounds in raspberry plants under progressive water deficit conditions across two growing seasons (2022-2024)**

| Growing Season | Treatment Level | Soluble Sugar Components | | | Primary Osmolyte |
| --- | --- | --- | --- | --- | --- |
|  |  | Sucrose (%) | Glucose (%) | Fructose (%) | Proline (μmol/g) |
| 2022 | 100% | 2.36±0.15 c | 2.56±0.12 c | 3.49±0.23 c | 0.029±0.003 c |
| *(Three-level gradient)* | 50% | 2.67±0.18 b | 3.12±0.15 b | 4.21±0.28 b | 0.120±0.012 b |
|  | PEG | 3.15±0.23 a | 4.25±0.23 a | 4.95±0.34 a | 1.104±0.089 a |
| 2024 | 100% | 3.74±0.18 a | 2.59±0.15 b | 3.15±0.18 b | 0.017±0.002 b |
| *(Two-level comparison)* | PEG | 4.36±0.25 a | 3.09±0.18 a | 4.01±0.25 a | 0.424±0.045 a |

Values represent means ± SE. Different letters within each year indicate significant differences (P ≤ 0.05, Tukey's HSD test).

**Table S5. Simple treatment effects on key physiological parameters in raspberry plants across two experimental years (2022-2024)**

| Parameter | 2022 (Year 1) | | 2024 (Year 2) | |
| --- | --- | --- | --- | --- |
|  | 100% | PEG | 100% | PEG |
| Sucrose Content (%) | 2.36±0.15 b | 3.15±0.23 a | 3.74±0.18 a | 4.36±0.25 a |
| Proline Content (μmol/g) | 0.029±0.003 b | 0.16±0.089 a | 0.017±0.002 b | 0.424±0.045 a |
| Abscisic Acid (ng/g) | 89.85±1.23 b | 95.73±1.67 a | 90.37±1.45 b | 98.69±1.89 a |

*Values represent means ± SE . Different letters within each year indicate significant differences (P ≤ 0.05, Tukey's HSD test). Note: Sucrose accumulation showed treatment response only in 2022, while proline and ABA responded consistently across both years.*

**Table S6. Cultivar-specific responses to water deficit treatment across two growing seasons: Temporal dynamics of genotype × treatment interactions in raspberry plants (2022-2024)**

| Year | Cultivar | Treatment | RWC (%) | Leaf Temp (°C) | Chlorophyll (µmol/m²) | Quantum Yield | Proline (μmol/g) |
| --- | --- | --- | --- | --- | --- | --- | --- |
| 2022 | Diamond  Jubilee | 100% | 83.50±2.34 b | 20.80±0.45 f | 33.57±1.12 ab | 0.79±0.01ᵃ | 0.037±0.004 de |
|  |  | 50% | 81.21±2.89 b | 21.10±0.52ᵉ | 36.43±1.18 ab | 0.78±0.01ᵃ | 0.175±0.012 b |
|  |  | PEG | 56.03±3.12 c | 22.23±0.52 e | 32.18±1.01 b | 0.74±0.02ᵇ | 0.114±0.012 c |
|  | Jade | 100% | 95.00±1.89 a | 20.23±0.41 f | 38.76±1.45 a | 0.79±0.01ᵃ | 0.021±0.003 e |
|  |  | 50% | 77.85±2.67 b | 21.00±0.48ᵉ | 37.91±1.23 a | 0.78±0.01ᵃ | 0.055±0.006 d |
|  |  | PEG | 48.15±2.89 d | 22.30±0.48 e | 31.20±1.08 b | 0.74±0.02ᵇ | 0.2095±0.089 a |
| 2024 | Diamond  Jubilee | 100% | 89.40±2.12 a | 28.85±0.67 b | 40.60±1.23 b | 0.74±0.01 a | 0.018±0.002 c |
|  |  | PEG | 32.38±3.45 e | 31.08±0.78 a | 39.23±1.01 b | 0.73±0.02 ab | 0.598±0.045 a |
|  | Jade | 100% | 88.32±1.98 a | 29.07±0.71 bc | 44.10±1.45 a | 0.67±0.02 bc | 0.015±0.002 c |
|  |  | PEG | 66.68±4.12 b | 30.17±0.69 ab | 40.17±1.12 b | 0.61±0.03 c | 0.250±0.023 b |

Values represent means ± SE. Different letters indicate significant differences across all treatment combinations (P ≤ 0.05, Tukey's HSD test). Only parameters with significant cultivar × treatment interactions are shown.
